# Deriving novel atrial fibrillation phenotypes using a tree-based artificial intelligence-enhanced electrocardiography approach

**DOI:** 10.1038/s41746-025-02159-z

**Published:** 2025-12-04

**Authors:** Mehak Gurnani, Konstantinos Patlatzoglou, Joseph Barker, Libor Pastika, Boroumand Zeidaabadi, Ibrahim Antoun, Riyaz Somani, G. Andre Ng, Paolo Inglese, Lara Curran, Declan O’Regan, Nicholas S. Peters, Daniel B. Kramer, Jonathan W. Waks, Arunashis Sau, Fu Siong Ng

**Affiliations:** 1 National Heart and Lung Institute, Imperial College London, London, UK; 2 Department of Cardiology, Imperial College Healthcare NHS Trust, London, UK; 3 Department of Cardiovascular Sciences, Leicester British Heart Foundation Centre of Research Excellence, Leicester National Institute for Health and Care Research Biomedical Research Centre, University of Leicester, Leicester, UK; 4 Department of Cardiology, Glenfield Hospital, University Hospitals of Leicester NHS Trust, Leicester, UK; 5 MRC Laboratory of Medical Sciences, Imperial College London, London, UK; 6 Richard A. and Susan F. Smith Center for Outcomes Research in Cardiology, Beth Israel Deaconess Medical Center, Harvard Medical School, Boston, MA USA; 7 Harvard-Thorndike Electrophysiology Institute, Beth Israel Deaconess Medical Center, Harvard Medical School, Boston, MA USA; 8 Department of Cardiology, Chelsea and Westminster Hospital NHS Foundation Trust, London, UK

**Keywords:** Atrial fibrillation, Machine learning

## Abstract

Atrial fibrillation (AF) is classically categorised by arrhythmia duration, but these subtypes have limitations in capturing mechanistic and prognostic diversity. A variational autoencoder, trained on >1.1M ECGs, extracted representative features, filtered for an AF cohort of 20,291 unique patients. These features were input into an unsupervised tree-based clustering method to map AF heterogeneity as a tree structure and identify phenogroups. Five phenogroups stratified by future disease risk were identified: (1) higher-risk AF; (2) highest-risk AF with heart failure (HF); (3) average paroxysmal AF; (4) lower-risk paroxysmal AF; and (5) higher-risk paroxysmal AF. The tree trajectory positioned individuals based on shared traits, emphasising explainability. Paroxysmal phenogroups 4 and 5 differed in risk and ventricular structure, with phenogroup 5 exhibiting more adverse features. Mixed AF phenogroup 2 reflected advanced AF with greater HF burden and mortality risk. This AI-ECG framework augments AF subtypes with a risk-based dimension, supporting personalised care.

## Introduction

Atrial fibrillation (AF) is the most common form of sustained cardiac arrhythmia, with its global prevalence and incidence increasing over the past decade^1^, contributing substantially to elevated morbidity and mortality. It is an established independent risk factor for ischaemic stroke and death, increasing the risk for stroke by five-fold and being associated with a higher risk of heart failure^2^.

AF has traditionally been classified based on duration, with paroxysmal, persistent, long-standing persistent, and permanent AF being viewed as distinct categories. However, the 2023 American Heart Association (AHA) guidelines^3^ refined AF classification to follow a stage-based classification, reflecting the progressive and variable nature of AF. While these categories are simplistic and capture some aspects of atrial remodelling, thus guiding interventions such as catheter ablation^4^, their ability to predict clinical outcomes remains debated^5–9^. Additionally, the heterogeneity of AF, stemming from genetic, environmental, and comorbidity influences, adds complexity that is not well captured by the current subtypes.

To address these limitations, several studies^10–20^ have employed machine learning-based clustering approaches to identify distinct subgroups among AF patients. These studies typically use traditional clustering methods based on clinical variables, including demographics, laboratory data, and cardiovascular history, but crucially neglect important electrophysiological information. It is therefore unclear whether these clusters relate directly to AF pathophysiology or simply capture general cardiovascular risk factors^21^. With the increasing availability of electrocardiograms (ECGs), utilising these signals within unsupervised methods may better account for AF-specific electrophysiological differences, aiding in the identification of distinct AF subtypes and electrophenotypes^4,21,22^.

In this study, we employed an unsupervised artificial intelligence-enhanced electrocardiography (AI-ECG) method to identify novel AF subgroups based on electrophysiological signals. Given the heterogeneous nature of AF, we use a tree-based dimensionality reduction method to model a two-dimensional trajectory representation of the underlying population variation^23^. We first derive representational ECG features using a variational autoencoder (VAE), followed by the application of dimensionality reduction via a tree-learning method, DDRTree^24^, to create this model. The tree allows for the exploration of a spectrum of phenotypes by projecting ECGs that represent mixed, average phenotypes within the tree core, and those with extreme, distinct traits towards the branch peripheries. This approach enables more granular phenogrouping of AF traits, based on the ECG, potentially leading to better characterisation of AF subgroups.

## Results

There were 1,163,401 available ECGs collected from 189,539 unique secondary care patients attending Beth Israel Deaconess Medical Centre (BIDMC; Boston, USA). Individuals with an AF diagnosis were selected (details in the “Methods” section), and the first recorded ECG post-diagnosis was used, resulting in 20,291 available ECGs from unique patients for deriving the main AF DDRTree described in this paper. Of these, 5621 ECGs were recorded in AF while 14,670 ECGs were in sinus rhythm. In addition to these 20,291 index ECGs, these patients contributed 338,242 further ECGs to the BIDMC dataset.

### Constructing the tree

To input interpretable AI-ECG features into the DDRTree algorithm, median beats from the full BIDMC cohort were used to first train a VAE^25^ in an unsupervised manner (details in the “Methods” section). This dimensionality reduction technique captured 51 axes of maximal variation as latent features with high accuracy, reaching a median Pearson’s *R* of 0.99 (Supplementary Fig. 1). Derived latent features were then filtered for the AF population within BIDMC as input to DDRTree; VAE reconstruction performance in this sub-population also performed similarly (Supplementary Fig. 2), hence representing the constituent parts of AF median beat ECGs. DDRTree is an unsupervised clustering method that performs dimensionality reduction with reversed graph embedding to project data as a low-dimensional tree structure with branches assigned as clusters. This approach was developed to model single cell trajectories, but it has been applied to clinical and ECG data to model other heterogeneous conditions like hypertrophic cardiomyopathy^23^, type 2 diabetes mellitus^26^ and broad QRS complex^27^. DDRTree positions ECGs across the constructed trajectory such that those in proximity share morphological traits, while those further apart display distinct, differentiated morphological traits. The algorithm mapped each point onto the tree, providing coordinates and assigning it to one of 27 sub-branches. To enhance statistical power for downstream analyses and focus on meaningful regions of the tree with sufficient sample size, we consolidated these sub-branches into larger, representative branches (phenotypes) based on spatial proximity, using a distance-based unsupervised clustering approach (see the “Methods” section).

**Fig. 1:**
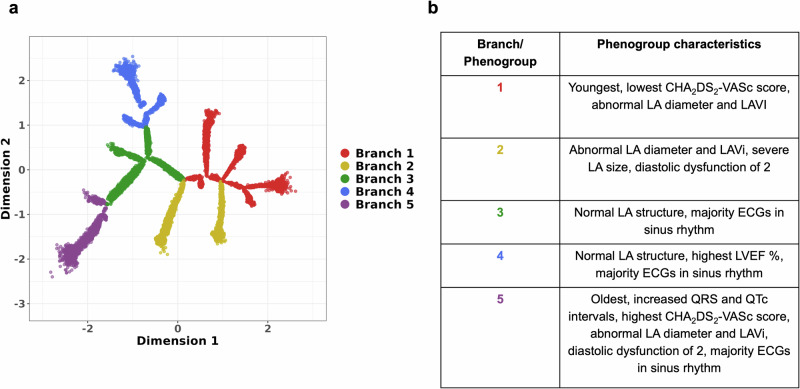
Deriving and characterising the BIDMC AF DDRTree. Subplot **a**) shows the resulting tree structure and branch assignments representing phenogroups from applying DDRTree to latent ECG features. Subplot **b**) shows the most distinctive traits seen across these phenogroups from descriptive analyses (refer to Supplementary Table 1 for greater detail). AF atrial fibrillation, BIDMC Beth Israel Deaconess Medical Centre, DDRTree dimensionality reduction via learning a tree, LA left atrial, LAVi left atrial volume indexed, LVEF left ventricular ejection fraction.

As a result, five main branches representing phenogroups were identified upon constructing the tree (Fig. 1a). We then characterised the tree dimensions and the phenogroups based on clinical variables, disease outcomes, echocardiography measures of cardiac structure and function and rhythm control therapies administered during follow-up.

### Description of the AF phenogroups

We first explored descriptive differences across the phenogroups; the key traits captured across each phenogroup are summarised in Fig. 1b (further details in Supplementary Table 1).

Phenogroup 1 comprised the youngest individuals, majority males with the lowest median CHA_2_DS_2_-VASc score of 2, with relatively increased left atrial (LA) size. Phenogroup 2 exhibited a comparable LA size to phenogroup 1, with notably greater diastolic dysfunction. Phenogroups 3-5 captured a majority of paroxysmal AF (PAF) phenotypes, with the index ECGs in sinus rhythm. Phenogroup 3 presented with average traits overall. Given its position in the tree core where mixed phenotypes are captured, it was considered the average baseline comparator for the remaining analyses. Phenogroup 4 was associated with having a more normal LA size and left ventricular (LV) function. Lastly, phenogroup 5 captured the oldest individuals within this population, with ECGs showing increased QRS duration and QTc intervals. It was also associated with the highest median CHA_2_DS_2_-VASc score of 4, abnormal LA size, and a similar diastolic dysfunction profile to phenogroup 2, but with an indication of underlying conduction disease. Overall, it was evident that various subtypes within AF were captured by DDRTree based on ECG features, each with distinct patterns and varying levels of structural dysfunction.

### Electrophysiology of the AF phenogroups and their association with latent ECG features

**Fig. 2:**
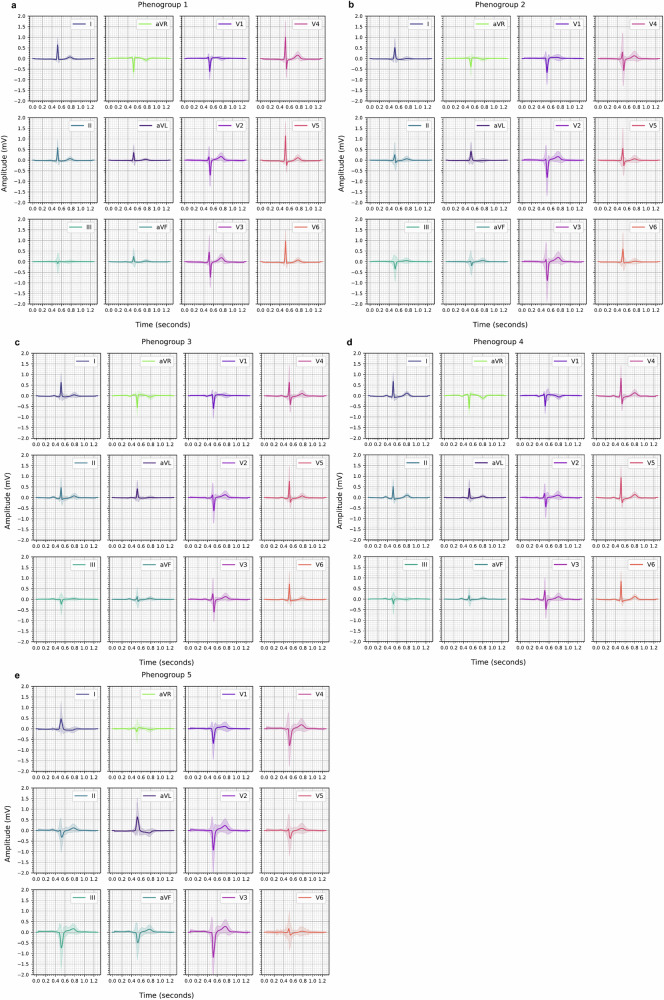
Representative median beat signals across the derived phenogroups in the BIDMC AF DDRTree. Subplots **a**–**e** show the averaged median beats using constituent ECGs for phenogroups 1–5 in a 12-lead format. For each lead, the solid line represents the median signal across all constituent median beat ECGs within the phenogroup, while the lighter shaded region indicates the variability of the signals, calculated as the standard deviation at each time point. AF atrial fibrillation, BIDMC Beth Israel Deaconess Medical Centre, DDRTree dimensionality reduction via learning a tree.

**Fig. 3:**
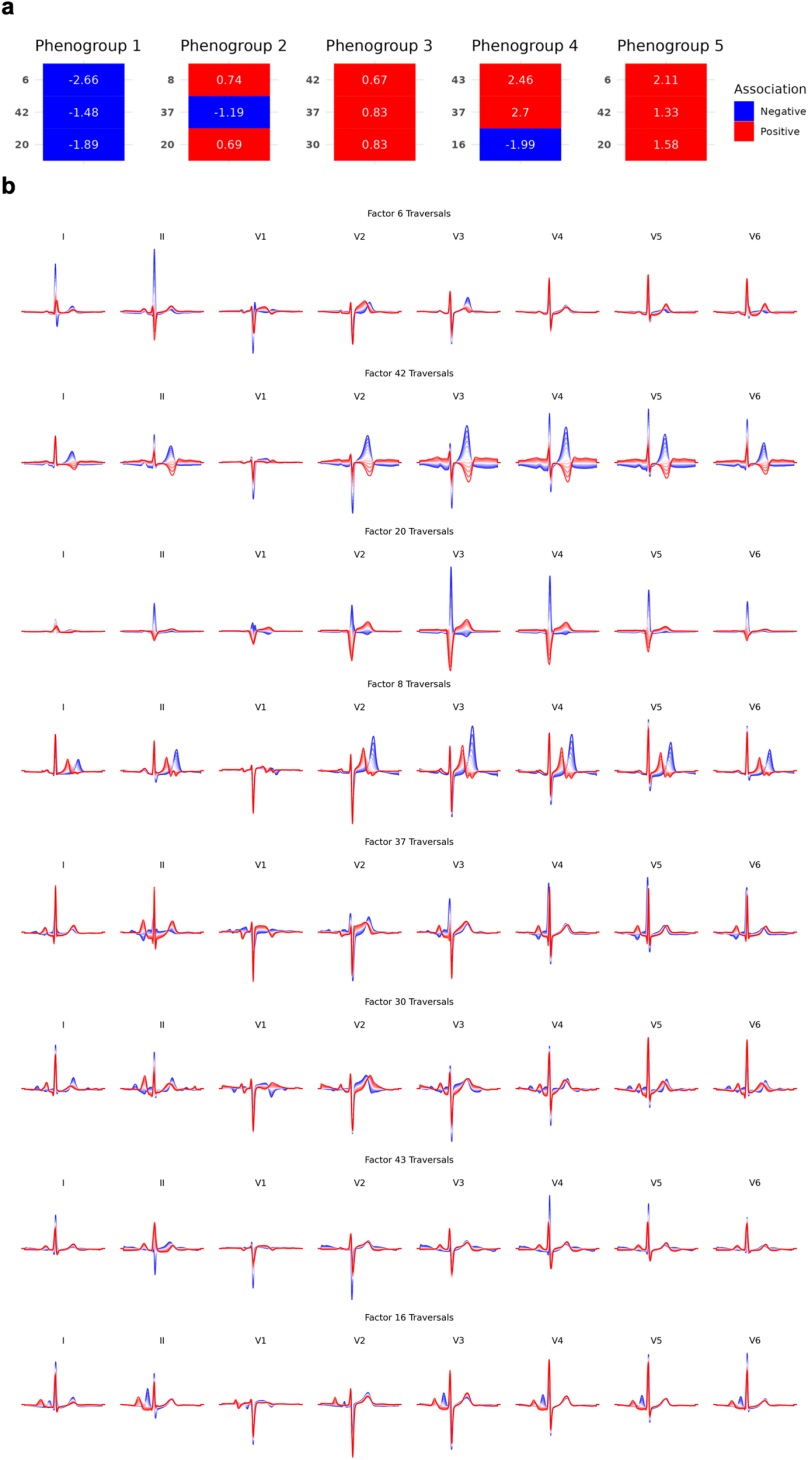
Explainability across BIDMC AF DDRTree phenogroups using latent ECG features. Subplot **a** shows the top 3 latent features for each phenogroup and the associated adjusted odds ratios. The estimates show the strength and direction of the association between the phenogroup and latent feature, when adjusted for all features. Subplot **b** shows the latent transversals for the selected latent ECG features in an 8-lead format. AF atrial fibrillation, BIDMC Beth Israel Deaconess Medical Centre, DDRTree dimensionality reduction via learning a tree.

We next visualised the averaged 12-lead median beat from constituent ECGs within each phenogroup (Fig. 2) to understand their ECG traits. Additionally, we used multivariate logistic regression models to regress the latent features against phenogroup assignments to identify the top 3 features associated with each phenogroup (Fig. 3a) and visualised the latent transversals^25^ of these selected features to provide further morphological insight (Fig. 3b).

Median beats from phenogroups 1 and 2 exhibited similar characteristics with narrow QRS complexes (Fig. 2). In contrast, representative signals from phenogroups 3 and 4 indicated that the majority of ECGs were in sinus rhythm, as evidenced by the presence of a P-wave, aligning with the previously observed PAF phenotype. Interestingly, phenogroups 1 and 5, at opposite ends of the tree, shared the same top 3 latent features (factors 6, 42 and 20) but with inverse associations, justifying the positions at opposite ends of the tree (Fig. 3a). Factor 42 reflected variations in T-wave morphology, while factor 20 captured differences in the QRS complex (Fig. 3a). Phenogroup 1’s negative association with both factors were indicative of normal T-wave morphology and a pronounced R peak in the QRS complex. In contrast, phenogroup 5 (majority paroxysmal AF) showed abnormal T-wave inversion in leads V2-V6 and an rS QRS pattern with deep S waves, potentially driven by an underlying bundle branch block, consistent with observations of abnormal QRS duration in this phenogroup (Fig. 2).

### Visual and spatial distribution of AF-traits across the tree

**Fig. 4:**
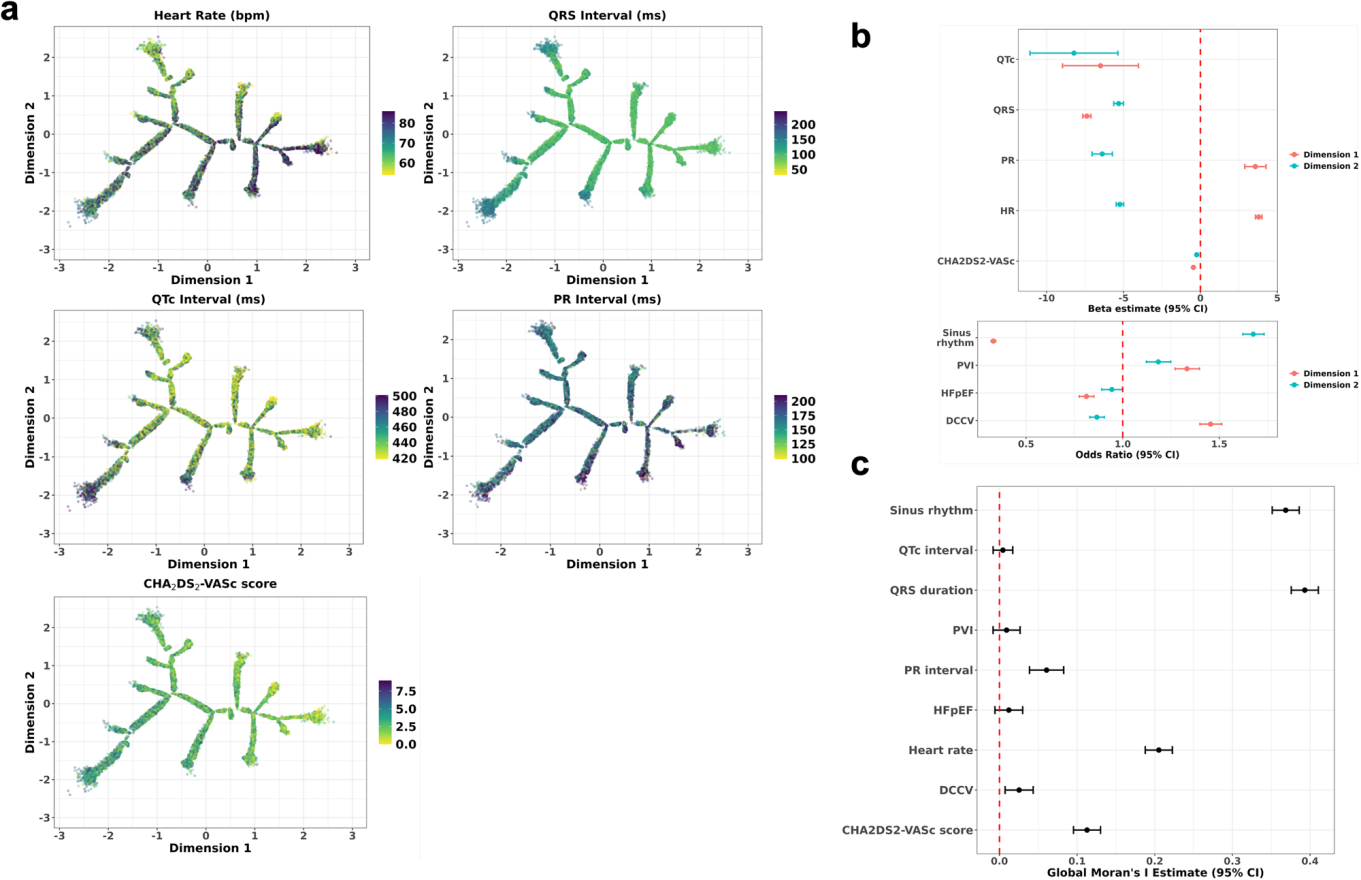
Distribution of AF-related phenotypes across the BIDMC AF DDRTree. Panel **a** shows the visual distributions of electrocardiography measures (heart rate, QRS, QTc, and PR interval) and CHA_2_DS_2_-VASc score across the tree. Panel **b** consists of forest plots showing the regression estimates (beta estimates/odds ratios) of the tree dimensions against the phenotypes. The red dotted line refers to a beta estimate of 0 or an exponentiated odds ratio of 1. Subplot **c** shows the resulting Global Moran’s *I* estimates and 95% confidence intervals across the phenotypes explored. AF atrial fibrillation, BIDMC Beth Israel Deaconess Medical Centre, DDRTree dimensionality reduction via learning a tree, DCCV direct current cardioversion, HFpEF heart failure with preserved ejection fraction, HR heart rate.

We then explored whether the tree captured underlying variation in AF-related phenotypes, including ECG measurements, clinical characteristics, and rhythm control therapies (AF ablation or direct current cardioversion (DCCV)). We visually characterised these trends by overlaying the phenotypes on the tree (Fig. 4a, Supplementary Fig. 3). These were then quantified using two approaches: regressing each phenotype against the tree dimensions to capture linear trends along the dimensions (Fig. 4b) and assessing the strength of spatial correlations using global Moran’s *I* values (Fig. 4c).

QRS duration, heart rate, and sinus rhythm showed the strongest spatial distributions (*p* < 0.0001). Greater values of QRS duration, QTc interval, and CHA_2_DS_2_-VASc score were captured within the bottom left region of the tree (i.e., low dimension 1 and low dimension 2), with higher values of PR interval (for ECGs in sinus rhythm) and heart rate were seen in the bottom right region.

In terms of rhythm control therapies, AF ablation labels did not exhibit significant spatial clustering, suggesting a more uniform distribution across the phenotypic space (Fig. 4c). However, when regressed against the tree dimensions, AF ablation was most frequently observed in the top right region (i.e., where both dimension 1 and dimension 2 are high) (Fig. 4b). In contrast, DCCV showed evidence of spatial autocorrelation, indicating that it tended to cluster within certain phenotypic profiles. Its highest frequency was observed in the bottom right region of the tree. These findings suggest distinct spatial patterns in the administration of rhythm control therapies during follow-up, along dimension 2, with AF ablation distributed more broadly and DCCV concentrated within specific clusters.

### Trends across prevalent cardiovascular disease burden

**Fig. 5:**
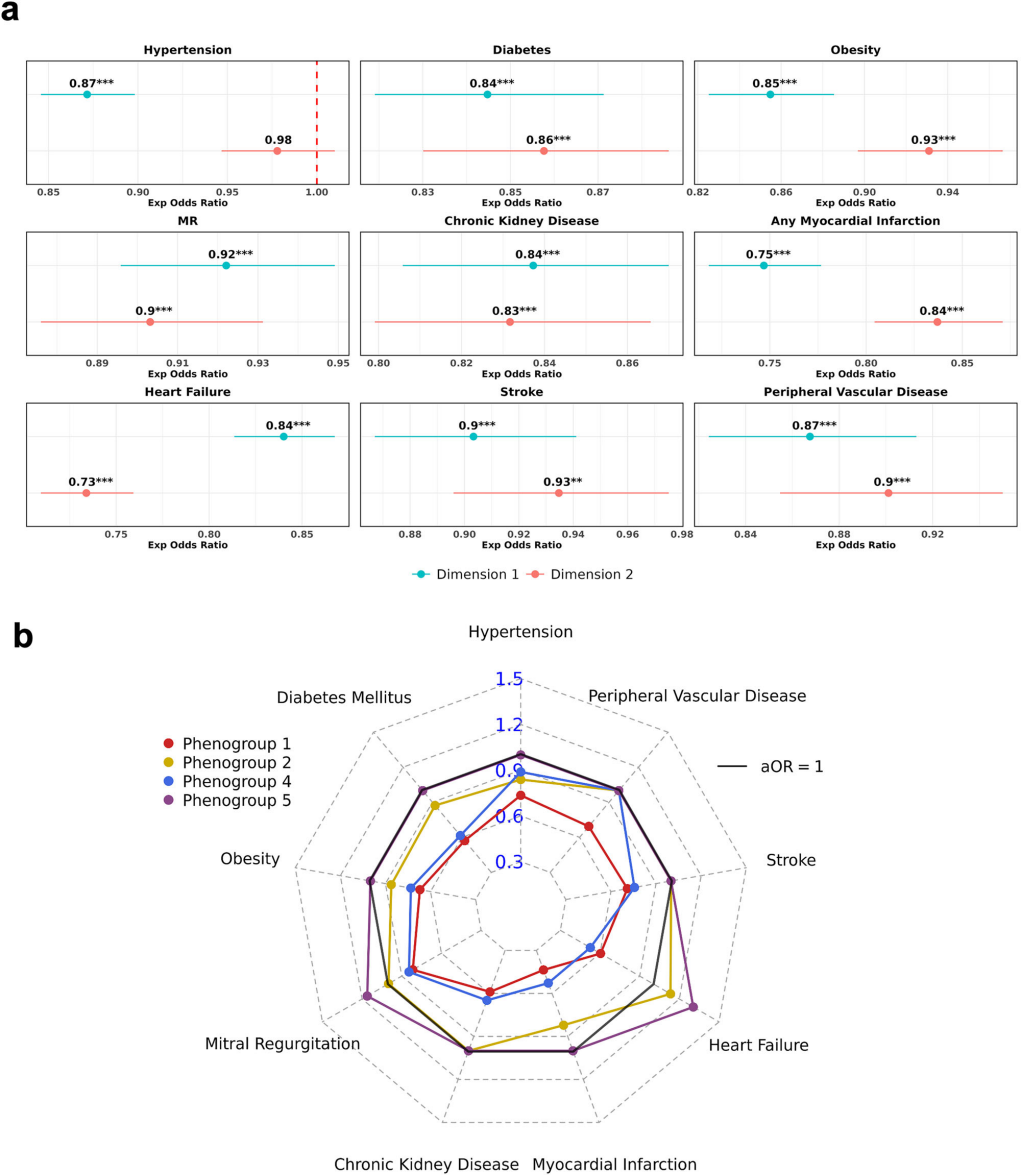
Investigating trends in prevalent cardiovascular disease burden across BIDMC AF DDRTree phenogroups. Panel **a** Forest plots showing the adjusted odds ratios (aOR) of tree dimensions against prevalent diseases, adjusted for covariates. The dotted red line indicates an aOR of 1, indicating non-significance. Subplot **b** is a radar plot showing the aORs across phenogroups for each prevalent disease, using phenogroup 3 as the baseline comparator. Regression estimates that did not reach statistical significance (*p*-value > 0.05) were set at an aOR of 1. All models presented were adjusted for age, sex, heart rate, QRS, and QTc interval. AF atrial fibrillation; BIDMC Beth Israel Deaconess Medical Centre; DDRTree dimensionality reduction via learning a tree; MR mitral regurgitation.

We next examined patterns of comorbidity burden across the tree by regressing the tree variables (dimension coordinates and phenogroup assignments) against prevalent disease outcomes using multivariate logistic regression models, adjusted for covariates. Full estimates from the prevalent analyses against phenogroups are provided in (Supplementary Table 2, Supplementary Fig. 4a). Moving along both dimensions 1 (rightward) and dimension 2 (upward) of the tree was consistently associated with decreased odds of prevalent disease (Fig. 5a), a trend that aligned with the phenogroup positions across the dimensions. Specifically, phenogroups 2 and 5, positioned lower in dimensions 1 and 2, respectively, were associated with increased odds of prevalent heart failure (HF) by 13% (adjusted odds ratio [aOR]: 1.13, 95% CI: 1.02–1.25, *p* < 0.05) and 31% (aOR: 1.31, 95% CI: 1.15–1.48, *p* < 0.0001) (Fig. 5b). Additionally, phenogroup 5 was associated with a 16% increase in mitral regurgitation burden (aOR: 1.16, 95% CI: 1.03–1.31, *p* < 0.05). In contrast, phenogroups 1 and 4, located further along dimensions 1 and 2, respectively, were both associated with lower odds of prevalent disease overall.

### Trends across incident cardiovascular disease and mortality outcomes

**Fig. 6:**
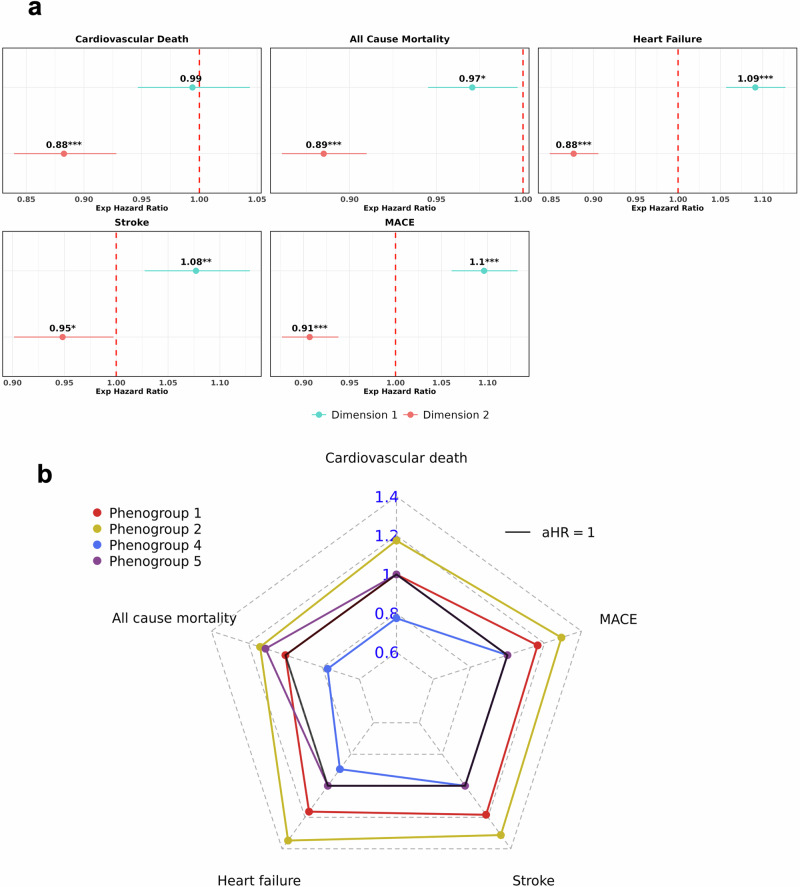
Investigating trends in incident cardiovascular disease and mortality outcomes across BIDMC AF DDRTree phenogroups. Panel **a**) Forest plots showing the adjusted hazard ratios (aHR) of tree dimensions against incident diseases, adjusted for covariates. The dotted red line indicates an aHR of 1, indicating non-significance. Subplot **b**) is a radar plot showing the aHRs across phenogroups for each incident disease, using phenogroup 3 as the baseline comparator. Regression estimates that did not reach statistical significance (*p*-value > 0.05) were set at an aHR of 1. All models were adjusted for age, sex, heart rate, QRS, QTc interval and CHA_2_DS_2_-VASc score. AF atrial fibrillation, BIDMC Beth Israel Deaconess Medical Centre, DDRTree dimensionality reduction via learning a tree, MACE major adverse cardiovascular events.

Similarly, we investigated the distribution of future disease risk across the tree using multivariate Cox Proportional Hazards models. Full estimates from the incident analyses against phenogroups are provided in (Supplementary Table 3, Supplementary Fig. 4b). Moving along dimension 2 of the tree (upward) was associated with significantly decreasing risk across all outcomes (Fig. 6a), aligning with phenogroup 4’s position further along dimension 2, which reflected a lower risk profile due to significantly decreased future risk of cardiovascular death, all-cause mortality, and HF (Fig. 6b).

Conversely, moving horizontally along dimension 1 (rightward) was significantly associated with 3% decrease in the risk of all-cause mortality (adjusted hazards ratio (aHR): 0.97, 95% CI: 0.95–0.99, *p* < 0.05) but an increased risk of HF, stroke and MACE by 9% (aHR: 1.09, 95% CI: 1.06–1.13, *p* < 0.0001), 8% (aHR: 1.08, 95% CI: 1.03–1.13, *p* < 0.01) and 10% (aHR: 1.10, 95% CI: 1.06–1.13, *p* < 0.0001), respectively (Fig. 6a). This pattern suggests that the highest risk of cardiovascular outcomes, including HF, stroke, and MACE, was concentrated in the bottom-right quadrant of the tree (high dimension 1, low dimension 2), where phenogroup 2 was located, while the highest risk of all-cause mortality appeared in the bottom-left quadrant, where phenogroup 5 was positioned. Phenogroup 2 was significantly associated with increased risk of future disease across all outcomes, with the highest hazards for future HF (aHR: 1.35, 95% CI: 1.21–1.50, *p* < 0.0001), compared to baseline (Fig. 6b). Meanwhile, phenogroup 5 experienced increased risk of future all-cause mortality by 11% (aHR: 1.11, 95% CI: 1–1.22, *p* < 0.05).

### Trends in cardiac structure and function

**Fig. 7:**
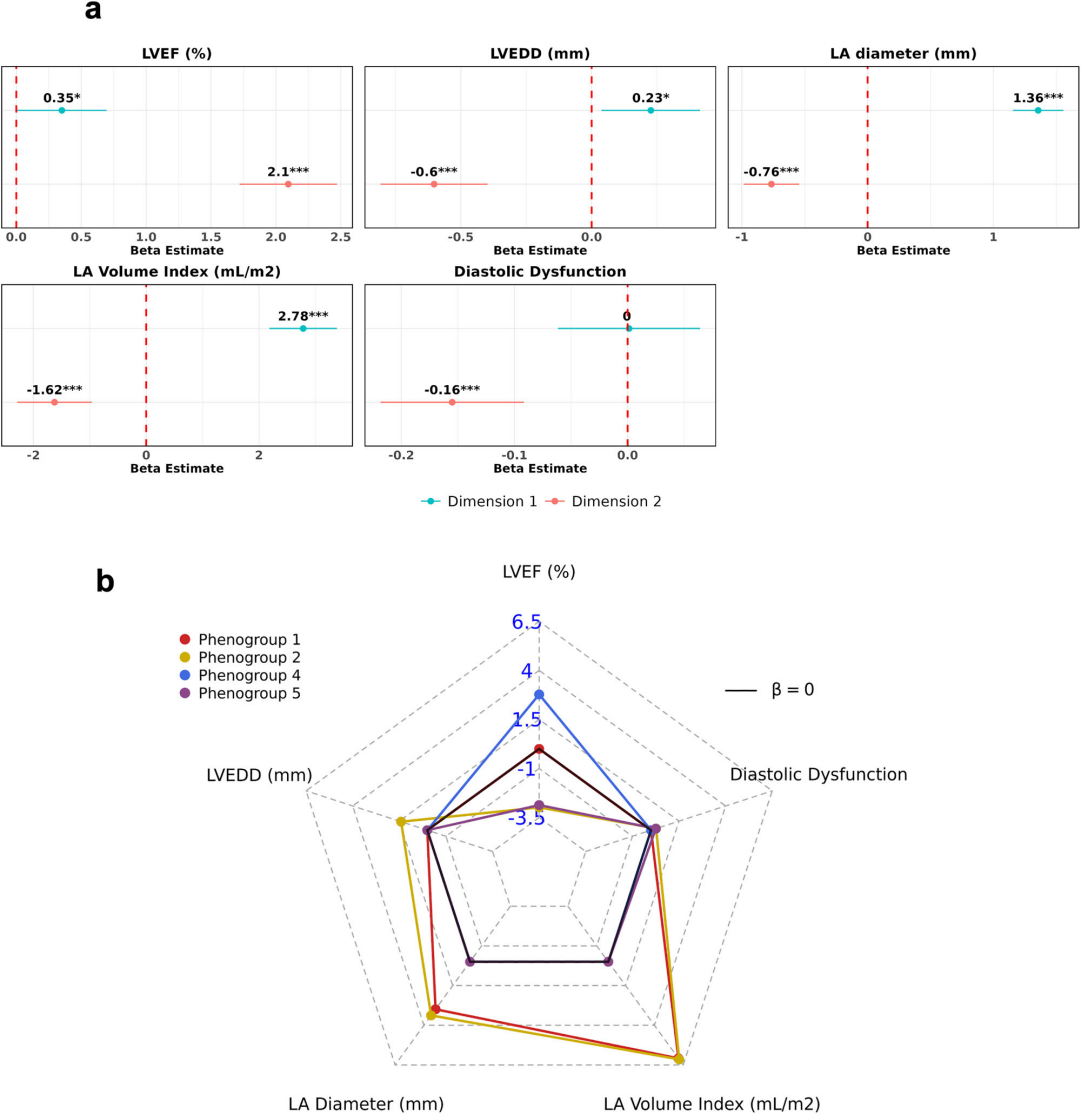
Trends across LV/LA structural and functional echocardiography measures in the BIDMC AF DDRTree. Panel **a** shows various forest plots with adjusted β estimates from regressing each of the tree dimensions 1 and 2 against echocardiography measures. The red dotted line refers to an adjusted *β* estimate of 0, indicating non-significance. Subplot **b** shows a radar plot of adjusted beta (*β*) estimates for each phenogroup against various measures, using phenogroup 3 as the baseline comparator. Regression estimates that did not reach statistical significance (*p*-value > 0.05) were set at a *β* of 0 within the plot. All models presented were adjusted for age, sex, heart rate, QRS, and QTc interval, and CHA_2_DS_2_-VASc score. AF atrial fibrillation, BIDMC Beth Israel Deaconess Medical Centre, DDRTree dimensionality reduction via learning a tree, LA left atrial, LV left ventricular ejection fraction, LVEDD left ventricular end diastolic diameter.

To assess trends in cardiac structure and function, we first overlaid relevant echocardiography measures across the tree (Supplementary Fig. 5). We then regressed the tree variables against these measures using multivariate linear regression models, adjusting for covariates (Fig. 7). Full estimates from the linear regression analysis against the phenogroup assignments are presented in (Supplementary Table 4, Supplementary Fig. 6).

Across the tree regions, the tree dimensions were differentially associated with the echocardiography measures. Moving along dimension 2 (vertically) was strongly associated with improving LV/LA size and function, indicated by increasing LV ejection fraction (LVEF) and decreasing LV end-diastolic diameter (LVEDD), LA diameter, LA indexed volume (LAVi), and diastolic dysfunction (Fig. 7a). Conversely, moving along dimension 1 (horizontally) was strongly associated with increasing LVEDD, LA diameter, and LAVi.

As a result, phenogroup 4, consisting of the majority of PAF individuals and located in the top-left quadrant, was associated with an increase in LVEF by 2.78% (1.54–4.01, *p* < 0.001) (Fig. 7b). In contrast, phenogroup 2, previously linked to individuals with a higher HF burden and located in the bottom-right region, captured those with worsening LV function and size. This was reflected by a decrease in LVEF of −2.98% per unit (95% CI: −4.12 to −1.84, *p* < 0.0001), an increase in LVEDD by 1.40 mm per unit (95% CI: 0.78–2.03, *p* < 0.0001), and an increase in diastolic dysfunction by 0.27 (95% CI: 0.07–0.48, *p* < 0.01) (Fig. 7b).

### Characterising DDRTree phenogroups against real-world AF management

To assess how the derived phenogroups align with established AF therapies, we investigated their association with rhythm control strategies administered during follow-up, including AF ablation and DCCV, using multivariate logistic models adjusted for covariates.

**Fig. 8:**
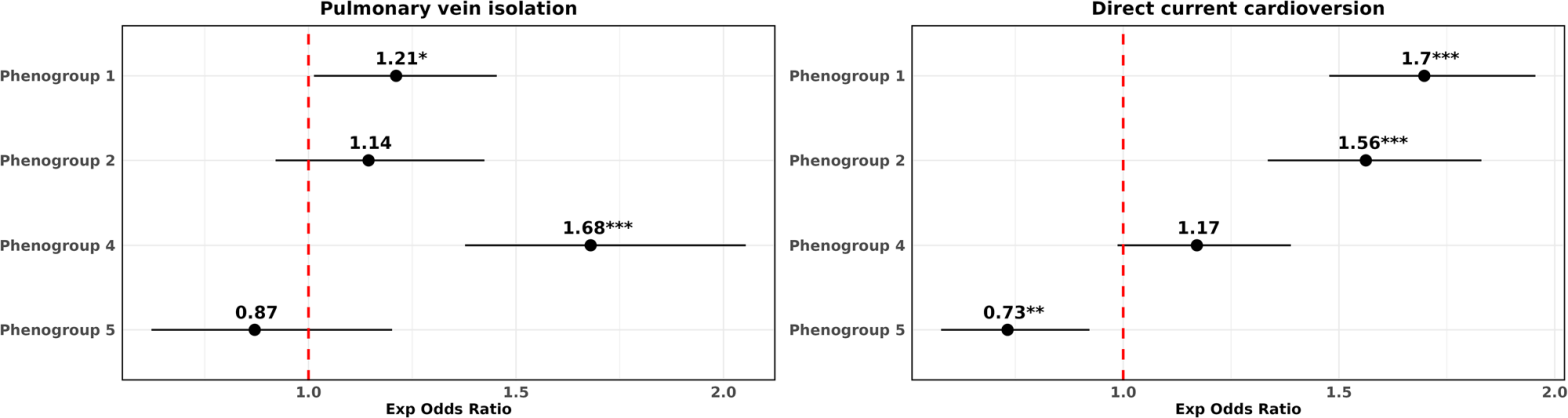
Trends in the administration of rhythm control treatments across BIDMC AF DDRTree phenogroups. Forest plots showing the adjusted odds ratios (aOR) from regressing the phenogroups against rhythm control treatments (pulmonary vein isolation or direct current cardioversion) administered post follow-up. The red dotted line refers to an aOR of 1, indicating non-significance. All models presented were adjusted for age, sex, heart rate, QRS, and QTc interval, and CHA_2_DS_2_-VASc score. AF atrial fibrillation; BIDMC Beth Israel Deaconess Medical Centre; DDRTree dimensionality reduction via learning a tree.

The low-risk phenogroup 4 showed a strong association with AF ablation, with significantly increased odds of receiving AF ablation during follow-up—68% greater odds compared to the baseline phenogroup (aOR: 1.68, 95% CI: 1.38–2.05; *p* < 0.0001), consistent with the predominantly PAF phenotype observed in this group (Fig. 8). While high-risk phenogroup 1 also showed a weaker association with increased odds of AF ablation (aOR: 1.21, 95% CI: 1.01–1.45, *p* < 0.05), individuals in this phenogroup were more strongly associated with increased odds of receiving DCCV (aOR: 1.70, 95% CI: 1.48–1.95, *p* < 0.0001), a pattern similarly observed in the proximal high-risk phenogroup 2. In contrast, individuals in phenogroup 5 were associated with significantly decreased odds of receiving DCCV (aOR: 0.73, 95% CI: 0.58–0.92, *p* < 0.01).

### Defining the phenogroups

**Fig. 9:**
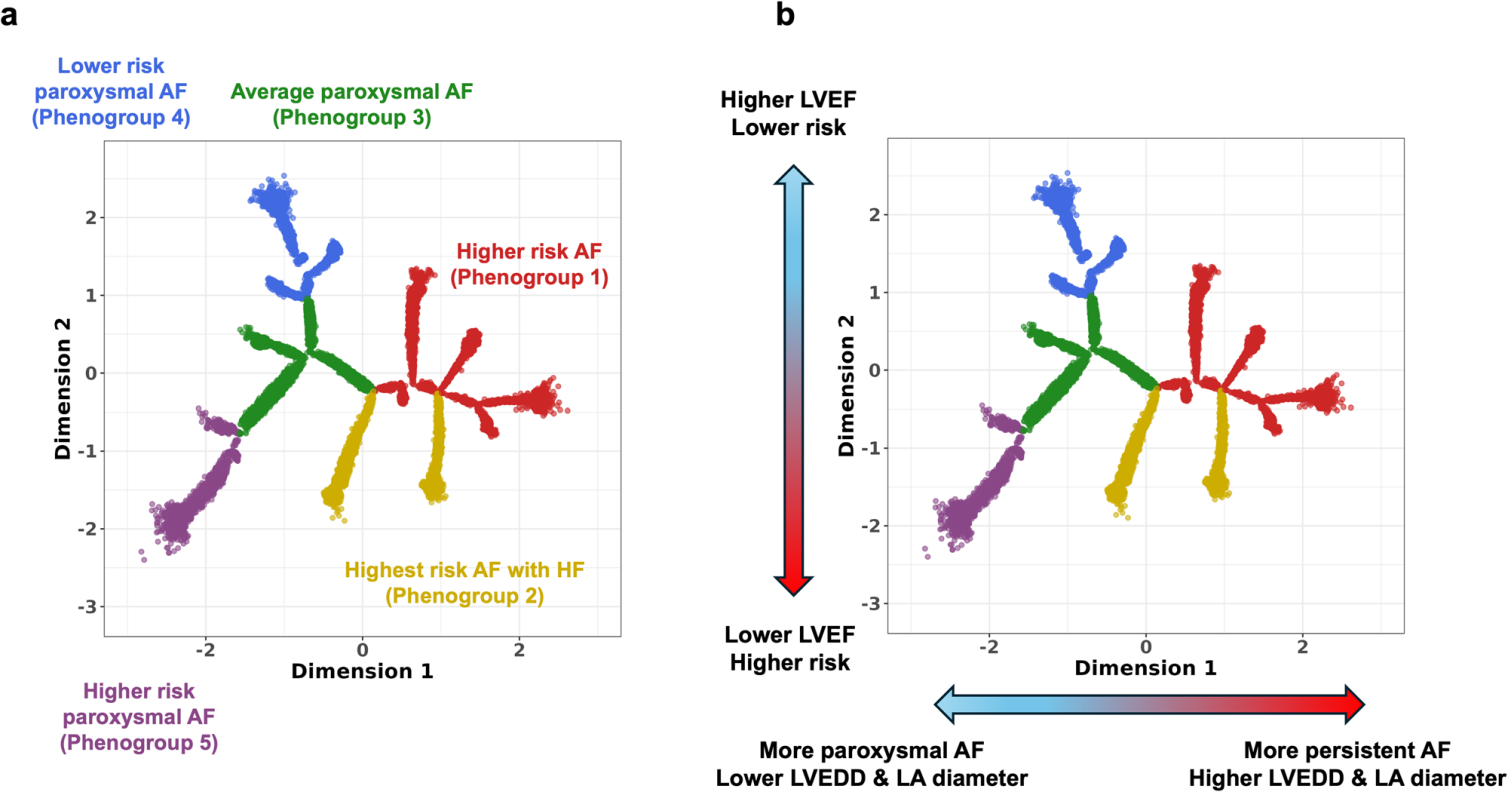
AF phenogroups derived using the DDRTree approach. Subplot **a** shows the five phenogroups identified within the BIDMC AF population, each labelled according to their disease risk profile and AF type. Distinct colours represent the different phenogroups. Subplot **b** illustrates the trends in AF type, future risk of disease (incident disease), and LV/LA function and size measures across the two main dimensions of the tree, aligning with the phenogroup profiles in each region. AF atrial fibrillation, BIDMC Beth Israel Deaconess Medical Centre, DDRTree dimensionality reduction via learning a tree, HF heart failure, LA left atrial, LVEDD left ventricular end-diastolic diameter, LVEF left ventricular ejection fraction.

Based on the above, we identified five phenogroups within the AF spectrum. These were labelled based on the phenogroups’ future risk of disease profile and dominant AF type. The following phenogroups were identified: (1) higher risk AF, (2) highest risk AF with HF, (3) average paroxysmal AF, (4) lower risk paroxysmal AF, and (5) higher risk paroxysmal AF as indicated by different colours in Fig. 9.

### Sensitivity analyses

We performed additional sensitivity analyses. First, when restricting to ECGs recorded near the initial AF diagnosis (within 60 days), the prevalent and incident disease profiles were consistent with the main analysis (Supplementary Fig. 7). Second, we assessed whether AF DDRTree phenogroups retained associations beyond current clinical and echocardiographic variables by restricting the analysis to ECGs linked to echocardiography performed within 60 days. We applied two levels of adjustment: (a) for age, sex, and CHA₂DS₂-VASc score (Supplementary Table 5), and (b) further adjusting for LVEF%, LA size, and AF/SR status at the time of recording (Supplementary Table 6). We found that associations across phenogroups 2, 4, and 5 were retained, with directionality consistent with previous results. This suggests that our phenogroups provide added prognostic data beyond conventional classification.

Rhythm at the time of recording was examined to verify its effect on phenogroup assignment. First, we assessed the association between rhythm status and phenogroup assignment within the BIDMC tree using Pearson’s correlation and found no strong correlations, suggesting that rhythm did not directly impact assignment (Supplementary Table 7). Second, to evaluate phenogroup assignment stability, we modified the selection criteria for cohort derivation to allow multiple ECGs per patient after AF diagnosis and analysed pairs of ECGs within one month where rhythm changed from AF-to-SR (5590 pairs) or SR-to-AF (2013 pairs). Changes were quantified using transition matrices (Supplementary Fig. 8).

We found that phenogroup assignment in AF-to-SR transitions was generally more stable, as indicated by the majority of values along the matrix diagonal, suggesting that most ECGs remained in the original phenogroup. This stability was particularly evident in the paroxysmal phenogroups 4 and 5, with 69.1% and 56% of ECGs, respectively, retained in their assignments (Supplementary Fig. 8a). Phenogroups 1 and 2 showed smaller shifts into neighbouring mixed branches such as phenogroup 3. In contrast, during SR-to-AF transitions, ECGs recorded in AF demonstrated lower stability, with most transitions across phenogroups more frequently re-assigned to the mixed AF phenogroups 1 and 2 (Supplementary Fig. 8b). Overall, these results suggest that while rhythm at the time of recording partially influences phenogroup stability, it does not affect overall phenogroup assignment.

### External validation

To externally validate this approach, the UK Biobank^28^ (UKB) cohort, which represents a healthy volunteer population^29^, was used. The VAE, trained on the full BIDMC cohort, was used to extract 51 representative latent features for the UKB population, comprising 65,162 available ECGs without additional training or fine-tuning. For the AF UKB subset (2344 ECGs), latent features were selected, and the population was projected onto the BIDMC-derived tree by predicting the tree coordinates and phenogroup assignments in a supervised manner (see the “Methods” section). The resulting arrangement of ECGs within the UKB tree closely mirrored the BIDMC tree (Supplementary Fig. 9a), with Spearman’s correlation coefficients showing similar distributions between the nearest points in both cohorts (Supplementary Fig. 9b).

The descriptive traits across the UKB-predicted phenogroups followed similar trends to those observed in the BIDMC AF phenogroups (Supplementary Table 8). We assessed the associations between UKB tree variables and disease outcomes, as well as CMR measures of LV and LA structure and function using regression-based models consistent with those applied in the BIDMC cohort. Trends in disease outcomes across UKB tree dimensions closely aligned with those in the BIDMC tree (Supplementary Fig. 10). Prevalent disease patterns in UKB phenogroups were largely consistent with BIDMC phenogroups, with two exceptions: phenogroup 2 displayed a significantly higher burden of chronic kidney disease (CKD), while phenogroup 5 showed an increased prevalence of both hypertension and CKD (Supplementary Table 9). Most associations for incident disease risk did not reach statistical significance, except for phenogroup 1’s association with future stroke risk (aHR: 4.78, 95% CI: 1.09–20.88, *p* < 0.05) (Supplementary Table 10).

Across CMR variables, UKB phenogroups 1 and 2 were similarly linked to impaired LV, LA function and increased LA size, compared to baseline phenogroup 3 (Supplementary Table 11). The main difference was in their association with diastolic dysfunction: only phenogroup 2 was significantly linked to worsening diastolic function, marked by increasing peak diastolic strain rate (PDSR) radial rates by 0.93 (95% CI: 0.48–1.38, *p* < 0.0001) and decreasing PDSR circumferential rates by −0.37 (95% CI: −0.55 to −0.2, *p* < 0.0001). Majorly, PAF phenogroups 4 and 5 also exhibited contrasting diastolic dysfunction patterns; phenogroup 4 was associated with improved diastolic function (increased longitudinal and circumferential PDSR rates and decreased radial rates), while phenogroup 5 showed the opposite trend, though without reaching statistical significance (Supplementary Table 11). Consistent with these trends, moving along dimension 1 was associated with worsening LV and LA size and function, while moving along dimension 2 was linked to improving measures (Supplementary Fig. 11).

## Discussion

In this study, we modelled and externally validated an AI-ECG-driven AF taxonomy using a combination of a generative VAE approach with an unsupervised clustering approach to explore underlying heterogeneity and identify novel AF phenogroups. As established in the guidelines, AF is a heterogeneous disease with an element of progression. Unlike other unsupervised clustering methods, DDRTree—originally developed for cell differentiation—has the advantage of intuitively capturing progression, making it well-suited for exploring AF heterogeneity in a novel way using ECG morphology features. Through this analysis, we identified five distinct phenogroups that exist within AF, each capturing unique characteristics of structural remodelling and future disease risk. Furthermore, our model demonstrated that the two-dimensional tree-based representation effectively captured meaningful trends across phenogroups, suggesting that the tree structure positioned phenogroups and individual ECGs in a clinically relevant manner. This approach offers advantages over simplistic clustering methods by assigning meaningful positions to clusters in the two-dimensional space.

Unlike traditional clustering approaches, our approach captured a spectrum of phenotypes. Our approach revealed two phenogroups (4 and 5) that captured predominantly paroxysmal AF patients, with differential comorbidity burden and future disease risk, identifiable from the ECG features. Phenogroup 4 represented a lower-risk AF subtype, with low prevalence of disease and normal LA/LV structure and function. Similar low-comorbidity phenogroups have been identified in other AF clustering studies^10,12–15^, including the UK Biobank cohort by Bellfield et al^20^. and may be consistent with clinically defined ‘lone’ AF. Phenogroup 5 instead captured a paroxysmal subtype with valvular heart disease (VHD), with the oldest subjects with a history of MR and HF. This may reflect shared risk factors, such as older age, for both AF and VHD.

We also identified two phenogroups (1 and 2) that contained predominantly persistent AF patients. In our DDRTree approach, positioning within each phenogroup provides additional information about comorbidity burden and disease risk. The spatial positioning along dimension 1 reflects this continuum, with dimension 1 being associated with increasing cardiovascular disease risk and increasing LA size. Phenogroup 2 was additionally associated with increased prevalent HF burden, mortality risk and diastolic dysfunction. The co-occurrence of AF and HF is known to worsen prognosis due to shared risk factors and pathophysiology^30,31^. This dynamic is further complicated by diastolic dysfunction, which is closely related to AF, and exacerbated by AF onset^32^. Thus, the elevated risk in this phenogroup likely stems from a complex interplay^33^ between AF, HF, and diastolic dysfunction, suggesting a more advanced atrial cardiomyopathy phenotype compared to phenogroup 1^34^.

We validated the phenogroups originally derived in the BIDMC population within the UKB cohort, with descriptive traits and trends in cardiac structure and function demonstrating strong alignment between the two cohorts. Discrepancies observed in prevalent and incident disease patterns were likely attributable to differences in cohort size and the healthy volunteer^29^ bias inherent to the UKB cohort, despite the overall consistency in the directionality of estimates. The observed asymmetry in phenogroup stability can be explained by the direction of rhythm change. In sinus rhythm, the P wave provides rich information on atrial remodelling and conduction, supporting differentiating between lower- and higher-risk paroxysmal groups (phenogroups 4 and 5). When transitioning to AF, this information is lost, and irregular R–R intervals with rate-dependent QRS/QTc can further obscure the difference, causing sinus-derived subgroups to collapse into mixed AF phenogroups. Conversely, transition back to sinus rhythm restores atrial conduction features and P-wave visibility, helping maintain the stability of phenogroup assignment. Clinically, these reassignments may reflect true disease progression, as AF promotes atrial remodelling and transition from paroxysmal to persistent forms. Thus, the clustering framework’s reclassification may capture meaningful shifts in phenotype and future disease risk.

Previous phenotyping studies have predominantly used baseline clinical data to derive clusters using methods such as hierarchical clustering^10–13,16–19^ and k-prototype clustering^14^. These methods, however, suffer from several limitations: (1) they neglect the electrophysiological signals in an electrophysiological disease, (2) they struggle to capture complex relationships within the data since they assume linear separability and can be sensitive to outliers, (3) AF is known for its heterogeneous nature, yet these methods assume homogeneity within clusters and clear distinctions between clusters. A recent study by Bellfield et al. ^20^ adressed these limitations by proposing a probabilistic ML approach to identify micro and macro clusters of AF patients. However, all these studies primarily use clinical data, meaning the clusters may not capture insights beyond what is already known to clinicians. By using novel AI-ECG representations, we overcome these limitations and capture phenogroups based on subtle morphological features not apparent to clinicians.

Our DDRTree approach shares some methodological similarities with the study by Bellfield et al.^20^. Both employ non-linear techniques to derive clusters after transforming high-dimensional features in a two-dimensional space, preserving the inherent structure of the data, and also model heterogeneity within clusters by identifying micro-clusters or sub-branches. This allows for the superimposition of additional variables not originally used for clustering, enhancing interpretability. However, DDRTree offers key advantages: it provides dimension coordinates alongside tree structure and phenogroup assignments, enabling a quantitative assessment of trends while accounting for the effect of covariates. Additionally, by using solely AI-ECG features to derive the tree rather than pre-defined clinical variables, these can additionally be used to project representative ECG signatures for the derived phenogroups, enhancing explainability and clinical relevance while taking a more personalised approach. While we currently explore our derived phenogroups further with a deeper phenotypic dataset, in future work, this approach could be further enhanced by integrating multimodal data sources—such as atrial structural remodelling, comorbidities, and patient background characteristics—to enable a deeper and more comprehensive personalised medicine strategy.

Given the characteristics of the phenogroups we identified, this ECG-based taxonomy indicates a classification of AF that could better capture mechanistic and prognostic diversity that could help guide personalised AF treatment strategies. For instance, the management of higher-risk phenogroups 1, 2, and 5 might differ based on their distinct traits. Our approach adds an additional dimension to the existing AF classification schema (paroxysmal, persistent, permanent). Building on current guidelines that prioritise early stroke risk assessment and timely intervention in AF management, our phenogrouping approach identified subjects who were associated with increased risks of stroke and heart failure. Phenogroup identification may guide risk management strategies and help personalise interventions. Higher risk groups may benefit from closer monitoring for heart failure, for example, or an aggressive rhythm control strategy, including AF ablation.

Results of the Early Treatment of Atrial Fibrillation for Stroke Prevention Trial (EAST-AFNET 4) trial^35^ have highlighted the importance of rhythm control in newly diagnosed AF, showing a reduced risk of cardiovascular outcomes. Further sub-analyses have demonstrated that early intervention retains its benefits in subsets of patients with prevalent heart failure burden^36^ as well as high comorbidity burden^37^. These findings raise the possibility that early intervention strategies might be particularly relevant for the higher-risk phenogroups identified in our study. Although we did not have data on post-treatment outcomes in the current study, it would be of interest to explore whether certain phenogroups, such as phenogroup 2 with its progressive features, may derive greater benefit from early rhythm control strategies, including AF ablation. However, these hypotheses would need to be formally evaluated in future prospective studies.

This study had several limitations. Although the inclusion criteria covered both disease codes and AF morphological features captured on ECG, there remains a small risk of inaccuracy due to potential miscoding, introducing the possibility of false positives or false negatives. There is a potential source of bias related to rhythm status at the time of ECG acquisition; the combined use of disease codes as well as ECGs, however, reduces this risk. In particular, AF vs sinus rhythm status may also cause changes in QRS morphology and QTc due to rate-related aberrancy. We aimed to mitigate this issue by adjusting for QRS duration and QTc in our regression analyses. Additionally, the known effect of medication use, particularly anti-arrhythmic therapy, on ECG features and consequently on phenogroup assignment, could not be tested due to unavailable data. Patients with a pacemaker implanted after an AF diagnosis or with postoperative pAF were not specifically excluded and likely represent a very small proportion of the cohort; while this may limit the applicability of our findings to these individuals, their inclusion is unlikely to have significantly influenced the overall results. Across the entire AF subset of BIDMC, we could not determine categories of persistent, long-standing persistent, and permanent AF or their trends across phenogroups. We lacked data on the outcomes post-AF ablation, hence we could not comment on how treatment success varied across the tree. Disease outcomes were defined using ICD-10 codes, which may lack granularity. ECG sampling, particularly from the BIDMC cohort due to its secondary care setting, may be subject to collider bias. The analysis was also constrained by the reliance on median beat ECG signals within the VAE, which may compress the beat-to-beat variability present within phenotypes. Furthermore, while visualising the latent transversals provides morphological insights into phenogroup traits, their biological interpretability is not straightforward, thereby complicating the linkage to clinical phenotypes. The BIDMC data were collected between 2000 and 2023, during which treatment practices likely changed. For example, there may be a reduction in digoxin-associated ST changes in newer ECGs. However, the broadly consistent findings in the UKB cohort which contains more recent ECGs (from 2014 to 2023) suggests this is unlikely to significantly affect our conclusions.

To conclude, we demonstrated the utility of an AI-ECG tree-based phenotyping approach to derive phenogroups in an unbiased manner, independent of clinical knowledge. Our analysis identified five phenogroups that, while sharing overlapping traits in terms of traditional AF classification, differ significantly in future disease risk and structural changes. In addition to validating this method in a separate external cohort, we highlight how our approach addresses the limitations of previous AF phenotyping studies and challenges the current oversimplistic clinical classification of AF.

## Methods

### Ethical approvals

This study complies with all relevant ethical regulations. The ethics review and approval for the Beth Israel Deaconess Medical Center (BIDMC) cohort were provided by the BIDMC Committee on Clinical Investigations (institutional review board protocol #2023P000042. Due to the retrospective nature of the BIDMC cohort, the requirement for individual patient consent was waived. The UK Biobank cohort received approval from the North West Multi-Centre Research Ethics Committee as a Research Tissue Bank (Application ID 48666). Individual participant informed consent was obtained in the UK Biobank.

### Cohorts

Beth Israel Deaconess Medical Centre (BIDMC) is a secondary care cohort composed of routinely collected data from Boston, USA. Individuals over 16 years old with valid ECGs collected between 2000-2023 were included. These resulted in 1,163,401 available ECGs from secondary care patients attending BIDMC. ECGs were linked to mortality records and other disease outcomes using ICD-9/10 codes. This cohort served as the derivation cohort to derive the AF DDRTree trajectory in this work.

The UK Biobank (UKB)^28^ cohort is a longitudinal study including over 500,000 volunteers aged between 40 and 79 at enrolment between 2006–2010 in the UK. This cohort represents a healthy volunteer population^29^ and was used for external validation within our study. It includes data from electronic health records, interviews and questionnaires conducted at baseline. A subset of UKB volunteers underwent further investigation using digital ECGs with available ECG measurements (*n* = 65,162). Outcomes were linked to death registry data, hospital admissions, cancer records, and primary care records. The UKB cohort served as the external validation cohort for the DDRTree approach implemented in the BIDMC cohort.

### Pre-processing of ECGs

ECGs available in a 12-lead format were pre-processed using a bandpass filter (0.5–100 Hz), notch filter (50/60 Hz, depending on the country of data acquisition), and resampling to 400 Hz. The BRAVEHEART ECG analysis software^38^ was used to create single median beats using the 8 leads from 10 s signals. Leads aVL, aVR, aVF and III were excluded since they can be derived from leads I and II.

### Variational autoencoder (VAE) model

A β-variational autoencoder (β-VAE) with a convolutional encoder/decoder architecture was trained in an unsupervised manner, as previously described and used for other AI-ECG tasks^39,40^. This model extends the standard autoencoder by incorporating a probabilistic latent space and introducing a Kullback–Leibler (KL) regularisation loss in addition to the reconstruction loss of the trained network. The model followed a convolutional autoencoder architecture: the encoder comprising six convolutional blocks, with a max pooling layer after every two blocks, and a final convolutional and fully connected layer, before the latent representation layer $z$. The decoder mirrored the encoder in a symmetrically inverse fashion. The encoder and decoder contained 1,533,888 and 1,283,976 parameters, respectively. *ReLU* activations were used in all layers. The model architecture is illustrated in Supplementary Fig. 12.

The β-VAE was trained on all available median beat ECGs from the BIDMC cohort using the annealed β-VAE^41^ objective. The dataset was split into training, validation, and test sets (85:5:10) with grouping applied by patient ID. No clinical data were utilised to derive these latent features; hence, this method followed an unsupervised approach. The reconstruction loss was set as the mean absolute error, and the KL loss was weighted by the *β* hyperparameter. The *β* hyperparameter controls the trade-off between reconstruction fidelity and disentanglement by limiting the information capacity of the latent units: higher *β* values increase the KL regularisation, encouraging the latent space to capture statistically independent factors of variation. In this study, *β* was set to 10 to ensure maximal latent feature disentanglement. An annealed training strategy was employed, progressively increasing the latent channel capacity (*C*) from 0 to a maximum value during training. This approach gradually relaxes the KL constraint, allowing the model to sequentially introduce more factors of variation while preserving previously learned independent factors. Model training was performed over 200 epochs using the Adam optimiser, with a batch size of 128 and an initial learning rate of 0.0005.

The initial number of latent dimensions explored was 256 to ensure sufficient model capacity. The number of latent features (51) was determined by the number of latent units with information capacity above 0.1 (as defined by KL divergence), which captured all meaningful variation in the data. This information capacity threshold was selected based on previous studies^42,43^. Maximal information capacity was also evident by reconstruction loss convergence (38.08 vs. 38.34 for the 51 informative factors). While testing the VAE, we used the *z*_mean_ representations of the ECG reconstruction and Pearson’s *R* as the performance metric.

The 51 latent features derived from the BIDMC cohort using the VAE model were used as input for DDRTree as described below.

### Selection criteria

Individuals with an AF diagnosis (determined by either an ICD code or an ECG recording of AF) were selected, and for each individual, the first recorded ECG post-diagnosis was used. AF was predicted from ECG recordings using a deep neural network (DNN) model^44^ across all cohorts in this study.

The BIDMC AF subset was used as the development cohort to derive the main AF DDRTree described in the paper. First, the 51 representative VAE-derived features were extracted for the BIDMC AF subset, then normalised and input into the DDRTree algorithm. We ran the DDRTree algorithm using default model parameters, except for the number of centroids. Different centroid values were evaluated to balance the tree complexity, stability of branch assignments, and computational demands. 2000 centroids were selected for the k-means component within each DDRTree iteration. The model output included DDRTree branch assignments, the coordinates for the tree dimensions (1 and 2), and pseudotime, a measure of distance from the tree center. Internal validation of the constructed tree achieved a median adjusted Rand Index of 0.55 (0.51–0.60).

The algorithm returned the dimension coordinates and sub-branch assignments for modelled data. Sub-branches were then consolidated into representative branches using hierarchical clustering, a distance-based unsupervised machine learning approach. Clusters were merged based on spatial proximity, using the normalised tree coordinates of the sub-branches as input. Since the coordinates were in a lower-dimensional space, we used Euclidean distance with an average linkage criterion to measure the distance between points. To determine the optimal number of clusters, we calculated silhouette scores at various heights of the dendrogram. The highest silhouette score, 0.44, indicated that the best clustering solution was achieved with five clusters. DDRTree was implemented using the 2.22.0 version of monocle^45^ from the Bioconductor repository.

### External validation

The UKB AF subset was used as the independent external validation cohort. This cohort was not utilised in the development of the BIDMC tree and was independently mapped onto the derived BIDMC AF DDRTree without further fine-tuning of the trajectory or clustering using DDRTree. First, the BIDMC-trained VAE model was used to extract representative latent features for the full UKB population, without further training or fine-tuning. The 51 latent features were selected for the AF subset within this cohort. To project the UKB AF cohort onto the BIDMC AF DDRTree, we then predicted their tree dimension coordinates and phenogroup assignments using supervised machine learning (ML) models trained on variables from the BIDMC AF DDRTree. No re-clustering or fine-tuning was applied, ensuring that the UKB cohort served as an independent external validation cohort projected directly onto the original BIDMC-derived tree. A two-step approach was followed to predict the coordinates (1) independent XGBoost regression models to predict each dimension’s coordinates, as performed by Curran et al.^23^. (2) A distance-estimating algorithm, as previously described by Nair et al.^26^. XGBoost models were trained on the VAE features from the BIDMC AF cohort using the dimension coordinates as the labels. A 75/25 train/validation split was applied, and the best-performing XGBoost parameters were identified through grid search for hyperparameter tuning. For both dimensions, hyperparameter tuning was performed using a five-fold cross-validation grid search on the training split. The hyperparameters explored included tree-specific settings such as max tree depth, subsample ratio, number of randomly selected features (colsample_bytree), number of trees (estimators), and the learning rate. The best-performing parameters for both dimension models were identical: a max depth of 5, subsample ratio of 0.6, colsample_bytree of 0.6, learning rate of 0.1, and 200 estimators.

These parameters were then used for training the models and to predict the dimension coordinates for the UKB AF cohort using their VAE features as input. The resulting validation split performance was *R*^2^_dimension 1_ = 0.86, mean absolute error (MAE_dimension 1_) = 0.35, *R*^2^_dimension 2_ = 0.89 and MAE_dimension 2_ = 0.27. Phenogroup assignments for the UKB AF population were predicted using a supervised *K*-nearest neighbour (KNN) model with the BIDMC AF tree coordinates as input to the model. 10-fold cross-validation was used to determine the optimal number of *K* neighbours (in a range of 1–200) based on the best performance on the internal holdout validation split using metrics like precision, recall, and F1-score, the harmonic mean of precision and recall. *K* of 1 achieved the best overall performance in the interval validation split (Supplementary Fig. 13).

### Outcome definition

ICD-9 and ICD-10 codes were used to define the presence of disease in both cohorts. Echocardiograms were available for a subset of BIDMC participants, while imaging instances within 60 days of an ECG were linked and used to assess associations of the tree variables to structural and functional changes. Within the UKB cohort, cardiac magnetic resonance imaging (cardiac MRI) data were available for a subset of the UKB cohort and were recorded on the same day as the ECG.

Rhythm control treatment data (AF ablation or DCCV) were available for the AF subset of the BIDMC cohort; however, post-treatment outcomes data were not available. No rhythm control treatment data were available for the UKB cohort.

### Statistical analysis

Within descriptive analyses, continuous values were shown as median values with interquartile ranges, while categorical data were expressed as frequencies and percentages. We quantified differences between phenogroups using the Kruskal-Wallis rank sum test for continuous variables and Pearson’s chi-squared test for categorical variables. In addition to overlaying variables on the tree, we regressed AF phenotypes against the tree dimension coordinates. We also assessed the spatial autocorrelation strength of these variables using Global Moran’s *I*. We quantified the associations between the tree variables (phenogroup assignments and tree dimensions) and echocardiographic measures using multivariate linear models, and the associations with prevalent disease outcomes and rhythm control treatments using multivariate logistic regression models. When assessing associations with rhythm control strategies, we excluded individuals who had received rhythm control treatments prior to the ECG recording. Similarly, for associations between tree variables and incident diseases, we excluded individuals who had a diagnosis of the disease prior to the subsequent ECG, ensuring the analysis focused on disease risk starting from the time of the ECG. For the analyses, we utilised multivariate time-to-event Cox proportional hazards models to assess the prognostic significance of the tree variables. Across all analyses, we adjusted for covariates such as age, sex, and electrocardiography measures (QRS duration, QTc interval, and heart rate) to isolate the underlying relationships between DDRTree-derived features and clinical outcomes and imaging endpoints. These adjustments account for well-established confounders in ECG and clinical analysis, highlighting the subtle, underlying relationships captured by the tree-based variables. We evaluated the risk of multicollinearity among these confounders using (1) correlation analyses across covariates and (2) variance inflation factors (VIF) calculations from multivariate logistic regression models against prevalent diseases in the BIDMC AF population. Since the phenogroup variable was modelled as a categorical predictor, the output included generalised VIF (GVIF) values. Based on prior studies^46,47^ that evaluated multicollinearity in the context of overlapping covariates, we used a tolerance threshold of 2.5–3 for scaled GVIF (i.e., GVIF adjusted for degrees of freedom). The resulting correlation and VIF results are found in Supplementary Fig. 14 and Supplementary Table 12, respectively. No evidence of multicollinearity was found. In the echocardiography, rhythm control treatment and incident disease analyses, adjustments were also made for the CHA_2_DS_2_-VASc score. We also conducted additional sensitivity analyses restricting the disease analyses to 1) ECGs within 60 days of the AF diagnosis to assess whether this modified the trends observed across the phenogroups and 2) ECGs linked to echocardiography performed within 60 days, adjusting additionally for LVEF %, LA size and AF/SR status to assess whether DDRTree phenogroups retained associations beyond current clinical standards. Additionally, we first assessed whether phenogroup assignment was primarily driven by rhythm status at the time of ECG acquisition, using Pearson’s correlation. Separately, we examined the effect of rhythm at the time of ECG recording on phenogroup assignment stability. This analysis was expanded from the original subset of unique AF patient ECGs (*n* = 20,291) to all ECGs recorded since AF diagnosis (*n* = 145,411). We then derived the AF/SR status and phenogroup assignment of these additional ECGs. AF/SR status was predicted using the DNN model^44^ previously mentioned, while phenogroup assignment was determined using our previously described external validation approach involving kNN models. Multiple ECG pairs per individual were then constructed to evaluate both AF-to-SR and SR-to-AF transitions within a defined timeframe, and changes in phenogroup assignment were quantified using transition matrices.

For the UKB cohort, we conducted similar analyses using cardiac MRI measures instead of echocardiography variables. For the disease analyses (prevalent and incident) across both cohorts, we aimed to investigate the same overlapping outcomes, but this was limited by what outcomes were available in the respective datasets.

Due to the high level of missingness (>30%) in electrocardiography variables in the UKB cohort, these measures were excluded as covariates in the UKB-specific analyses to preserve statistical power and ensure a sufficient sample size. We refrained from testing for proportional hazards in line with recent findings that suggest the adequacy of well-powered clinical datasets^48^. Considering these findings, we regarded the Cox model’s hazard ratio as an average estimate across the follow-up duration. All statistical analyses and hierarchical clustering models were built using R (version 4.1.3), while the variational autoencoder and supervised ML models were built using Python (version 3.9).

### Explainability

To visualise the representative electrocardiography across phenogroups, we plotted the average median beats per phenogroup in a 12-lead format. Additionally, to assess how the VAE latent features used to derive the BIDMC AF DDRTree correlated with the phenogroups, we used multivariate logistic regression models to regress the 51 representative features against each phenogroup. This enabled us to identify the top three most significant features while accounting for the impact of other features and their associations with the phenogroup. We visualised the latent transversals^25^ of the selected features to provide further morphological insight. A step size of 0.5 was used to visualise the latent traversals over a range of −3 to 3.

## Supplementary information

Supplementary material

## Data Availability

UK Biobank data are available upon application (http://www.ukbiobank.ac.uk/). The BIDMC dataset is restricted due to ethical limitations.

## Code availability

The code to create the DDRTree structure and project other data onto the tree is available at this link (https://github.com/mekgurnani/AF_DDRTree).
