# Supplementary material for: Deriving novel atrial fibrillation phenotypes using a tree-based artificial intelligence-enhanced electrocardiography approach
Source: NPJ Digit Med. 2025 Dec 4;8:779. doi: 10.1038/s41746-025-02159-z (PMC12722375; doi:10.1038/s41746-025-02159-z)
Supplement: Supplementary file 1 — Supplementary material [file 41746_2025_2159_MOESM1_ESM.pdf]

Supplementary material

Supplementary Table 1

Variable	Phenogroup 1, N = 7,346[1]	Phenogroup 2, N = 3,565[1]	Phenogroup 3, N = 4,025[1]	Phenogroup 4, N = 3,431[1]	Phenogroup 5, N = 1,924[1]	p-value[2]
**Baseline variables**						
**Age**	67 (17)	73 (17)	70 (17)	69 (16)	76 (17)	<0.001
**Females**	1,952 (27%)	1,004 (28%)	2,228 (55%)	1,906 (56%)	891 (46%)	<0.001
**Ethnicity**						<0.001
Asian	279 (3.8%)	87 (2.4%)	113 (2.8%)	128 (3.7%)	44 (2.3%)	
Black	516 (7.0%)	351 (9.8%)	362 (9.0%)	196 (5.7%)	193 (10%)	
Hispanic	222 (3.0%)	116 (3.3%)	128 (3.2%)	75 (2.2%)	68 (3.5%)	
Other	277 (3.8%)	162 (4.5%)	129 (3.2%)	120 (3.5%)	59 (3.1%)	
Unknown	301 (4.1%)	158 (4.4%)	118 (2.9%)	113 (3.3%)	76 (4.0%)	
White	5,751 (78%)	2,691 (75%)	3,175 (79%)	2,799 (82%)	1,484 (77%)	
**BMI**	28 (8)	28 (8)	29 (8)	28 (8)	28 (8)	0.031
Unknown	4,451	2,048	2,308	2,027	1,144	
**Systolic Blood Pressure (mmHg)**	130 (19)	129 (19)	130 (19)	130 (19)	130 (20)	0.59
Unknown	2,334	954	973	942	479	
**Diastolic Blood Pressure (mmHg)**	75 (12)	72 (12)	72 (12)	73 (12)	70 (12)	<0.001
Unknown	2,335	955	974	943	479	
**ECG measures**						
**Heart Rate**	72 (28)	75 (24)	69 (18)	62 (13)	73 (18)	<0.001
Unknown	3	1	4	3	5	
**QRS duration**	92 (16)	100 (28)	92 (20)	96 (30)	132 (52)	<0.001
Unknown	3	2	4	3	5	
**QTc interval**	434 (45)	447 (48)	438 (41)	435 (40)	467 (51)	<0.001
Unknown	1	0	1	0	2	
**PR interval**	168 (48)	184 (62)	166 (36)	164 (32)	172 (40)	<0.001
Unknown	3,907	1,966	586	340	700	
**Body surface area (m^2)**	2.01 (0.36)	1.99 (0.36)	1.91 (0.38)	1.90 (0.37)	1.91 (0.36)	<0.001
Unknown	2,083	803	921	897	370	
**Echocardiography measures**						
**LA diameter (mm)**	43 (11)	44 (11)	40 (10)	40 (10)	42 (10)	<0.001
Unknown	5,492	2,530	2,940	2,655	1,368	
**LA size**						<0.001
Mild	1,344 (35%)	711 (35%)	772 (33%)	614 (31%)	427 (37%)	
Moderate	712 (18%)	432 (21%)	279 (12%)	267 (14%)	208 (18%)	
Normal	1,413 (36%)	593 (29%)	1,141 (48%)	953 (49%)	378 (33%)	

Severe	419 (11%)	285 (14%)	163 (6.9%)	119 (6.1%)	144 (12%)	
Unknown	3,458	1,544	1,670	1,478	767	
**LA volume index (ml/m^2)**	35 (18)	38 (19)	32 (18)	32 (16)	37 (19)	<0.001
Unknown	6,530	3,069	3,575	3,100	1,658	
**LVEF (%)**	60 (15)	55 (22)	60 (18)	62 (14)	55 (25)	<0.001
Unknown	5,046	2,295	2,757	2,531	1,257	
**LVEDD (mm)**	46 (9)	48 (12)	45 (10)	45 (9)	48 (12)	<0.001
Unknown	5,477	2,507	2,915	2,645	1,344	
**Diastolic Dysfunction**	1.00 (1.00)	2.00 (2.00)	1.00 (1.00)	1.00 (1.00)	2.00 (2.00)	<0.001
Unknown	7,060	3,393	3,776	3,244	1,797	
**CHA_2DS_2-VASc score**	2 (3)	3 (3)	3 (3)	3 (2)	4 (2)	<0.001
**Days from AF**	12 (280)	22 (333)	42 (510)	37 (491)	51 (517)	<0.001
**Sinus Rhythm**	3,931 (54%)	2,057 (58%)	3,703 (92%)	3,219 (94%)	1,760 (91%)	<0.001
**Left Axis Deviation**	455 (6.2%)	1,157 (33%)	670 (17%)	320 (9.3%)	1,107 (58%)	<0.001
Unknown	6	8	7	6	23	
**Tobacco consumption**						0.004
False	7,112 (97%)	3,426 (96%)	3,844 (96%)	3,313 (97%)	1,842 (96%)	
True	234 (3.2%)	139 (3.9%)	181 (4.5%)	118 (3.4%)	82 (4.3%)	
**Alcohol consumption**	366 (5.0%)	214 (6.0%)	250 (6.2%)	167 (4.9%)	105 (5.5%)	0.017
**Prevalent disease**						
**Mitral Regurgitation**	2,391 (33%)	1,441 (40%)	1,612 (40%)	1,273 (37%)	892 (46%)	<0.001
**Hypertension**	4,385 (60%)	2,384 (67%)	2,788 (69%)	2,272 (66%)	1,416 (74%)	<0.001
**Diabetes Mellitus**	1,709 (23%)	1,227 (34%)	1,442 (36%)	867 (25%)	780 (41%)	<0.001
**Obesity**	1,249 (17%)	733 (21%)	968 (24%)	640 (19%)	478 (25%)	<0.001
**Chronic Kidney Disease**	857 (12%)	791 (22%)	766 (19%)	448 (13%)	534 (28%)	<0.001
**Myocardial Infarction**	761 (10%)	729 (20%)	825 (20%)	440 (13%)	540 (28%)	<0.001
**Peripheral Vascular Disease**	437 (5.9%)	376 (11%)	372 (9.2%)	271 (7.9%)	237 (12%)	<0.001
**Heart Failure**	1,482 (20%)	1,404 (39%)	1,326 (33%)	730 (21%)	1,037 (54%)	<0.001
**Stroke**	803 (11%)	550 (15%)	677 (17%)	461 (13%)	343 (18%)	<0.001
**Mortality outcomes**						
**All-cause mortality**	1,696 (23%)	1,256 (35%)	1,141 (28%)	722 (21%)	828 (43%)	<0.001
**Cardiovascular death**	480 (6.5%)	378 (11%)	329 (8.2%)	200 (5.8%)	275 (14%)	<0.001
**Rhythm control therapy**						
**Pulmonary vein isolation**	260 (3.5%)	84 (2.4%)	125 (3.1%)	135 (3.9%)	28 (1.5%)	<0.001
**Direct current cardioversion**	655 (8.9%)	283 (7.9%)	177 (4.4%)	126 (3.7%)	113 (5.9%)	<0.001

[1] Median (IQR); n (%)

[2] Kruskal-Wallis rank sum test; Pearson's Chi-squared test

Descriptive statistics of the BIDMC AF DDRTree phenogroups across baseline variables, echocardiography measures and disease outcomes. Continuous variables are shown as median values with the associated interquartile ranges and categorical variables are shown with their frequencies. Differences across phenogroups were quantified using the non-parametric Kruskal-Wallis rank sum test and Pearson's Chi-squared test for continuous and categorical variables, respectively using a p-value significance threshold of 0.05. Unknown values refer to missing values for variables.

AF atrial fibrillation; BIDMC Beth Israel Deaconess Medical Centre; BMI body mass index; DDRTree dimensionality reduction via learning a tree; LA left atrial; LVEDD left ventricular end-diastolic diameter; LVEF left ventricular ejection fraction.

Supplementary Table 2

Phenogroup	HTN	DM	Obesity	MR	CKD	MI	HF	Stroke	PVD
1	**0.74 (0.67-0.80), p < 0 .0001**	**0.57 (0.52-0.62), p < 0 .0001**	**0.67 (0.61-0.74), p < 0 .0001**	**0.82 (0.75-0.89), p < 0 .0001**	**0.59 (0.53-0.66), p < 0 .0001**	**0.44 (0.39-0.49), p < 0 .0001**	**0.60 (0.55-0.66), p < 0 .0001**	**0.71 (0.63-0.80), p < 0 .0001**	**0.69 (0.60-0.81), p < 0 .0001**
2	**0.84 (0.76-0.93), p < 0 .001**	**0.87 (0.79-0.96), p < 0 .01**	**0.86 (0.77-0.96), p < 0 .01**	1.08 (0.98-1.19), 0.11	1.01 (0.90-1.14), 0.87	**0.82 (0.73-0.92), p < 0 .01**	**1.13 (1.02-1.25), p < 0 .05**	0.92 (0.81-1.04), 0.19	1.09 (0.93-1.27), 0.31
4	**0.89 (0.80-0.98), p < 0 .05**	**0.62 (0.56-0.68), p < 0 .0001**	**0.73 (0.65-0.82), p < 0 .0001**	**0.85 (0.77-0.93), p < 0 .001**	**0.65 (0.57-0.74), p < 0 .0001**	**0.53 (0.46-0.60), p < 0 .0001**	**0.53 (0.47-0.59), p < 0 .0001**	**0.76 (0.66-0.86), p < 0 .0001**	0.86 (0.73-1.01), 0.07
5	1.06 (0.93-1.21), 0.39	1.03 (0.91-1.17), 0.59	1.02 (0.89-1.17), 0.77	**1.16 (1.03-1.31), p < 0 .05**	1.14 (0.99-1.31), 0.07	1.07 (0.93-1.24), 0.32	**1.31 (1.15-1.48), p < 0 .0001**	0.94 (0.80-1.10), 0.43	1.12 (0.93-1.36), 0.23

Adjusted odds ratio estimates from multivariate logistic models evaluating the associations between BIDMC AF phenogroups and prevalent disease outcomes. The adjusted odds ratios (aORs), 95% confidence intervals and associated p-values are presented for each phenogroup, using phenogroup 3 as the baseline comparator. All analyses were adjusted for age, sex and ECG measurements (heart rate, QRS duration, QTc interval).

AF atrial fibrillation; BIDMC Beth Israel Deaconess Medical Centre; CKD chronic kidney disease; DM diabetes mellitus; HF heart failure; HTN hypertension; MI myocardial infarction; MR mitral regurgitation; PVD peripheral vascular disease.

Supplementary Table 3

Phenogroup	CV death	All-cause mortality	Heart failure	Stroke	MACE
1	1.01 (0.87-1.17), 0.93	0.93 (0.86-1.01), 0.08	**1.16 (1.06-1.28), p < 0.01**	**1.18 (1.03-1.36), p < 0.05**	**1.16 (1.06-1.28), p < 0.01**
2	**1.17 (1.01-1.37), p < 0.05**	**1.14 (1.05-1.24), p < 0.01**	**1.35 (1.21-1.50), p < 0.0001**	**1.31 (1.12-1.53), p < 0.001**	**1.29 (1.16-1.44), p < 0.0001**
4	**0.78 (0.65-0.93), p < 0.01**	**0.77 (0.70-0.85), p < 0.0001**	**0.89 (0.80-1.00), p < 0.05**	1.02 (0.87-1.20), 0.79	0.92 (0.83-1.03), 0.16
5	1.15 (0.97-1.37), 0.12	**1.11 (1.00-1.22), p < 0.05**	1.13 (0.98-1.29), 0.09	1.04 (0.85-1.27), 0.73	1.04 (0.90-1.21), 0.56

Adjusted hazards ratio estimates from multivariate Cox proportional hazards models evaluating the associations between BIDMC AF phenogroups and incident cardiovascular disease and mortality outcomes. The adjusted hazards ratios (aHRs), 95% confidence intervals and associated p-values are presented for each phenogroup, using phenogroup 3 as the baseline comparator. All analyses were adjusted for age, sex and ECG measurements (heart rate, QRS duration, QTc interval) and CHA_2DS_2-VASc score.

AF atrial fibrillation; BIDMC Beth Israel Deaconess Medical Centre; CV cardiovascular; MACE major adverse cardiovascular events.

Supplementary Table 4

Phenogroup	LVEF (%)	LVEDD (mm)	LA diameter (mm)	LAVi (ml/m^2)	Diastolic dysfunction
1	0.70 (-0.32 to 1.72), 0.18	0.27 (-0.28 to 0.83), 0.34	**2.99 (2.40 to 3.58), p < 0.0001**	**6.09 (4.25 to 7.92), p < 0.0001**	0.02 (-0.16 to 0.20), 0.82
2	**-2.98 (-4.12 to -1.84), p < 0.0001**	**1.40 (0.78 to 2.03), p < 0.0001**	**3.38 (2.72 to 4.04), p < 0.0001**	**6.15 (4.13 to 8.17), p < 0.0001**	**0.27 (0.07 to 0.48), p < 0.01**
4	**2.78 (1.54 to 4.01), p < 0.0001**	-0.57 (-1.24 to 0.10), 0.09	0.30 (-0.41 to 1.01), 0.41	-0.80 (-3.01 to 1.41), 0.48	-0.17 (-0.36 to 0.03), 0.10
5	**-2.87 (-4.30 to -1.43), p < 0.0001**	0.65 (-0.13 to 1.43), 0.10	0.18 (-0.65 to 1.01), 0.67	0.43 (-2.07 to 2.93), 0.74	**0.26 (0.02 to 0.49), p < 0.05**

Adjusted beta estimates from multivariate linear models evaluating the associations between BIDMC AF phenogroups and echocardiography measures of LV/LA structure and function. The adjusted beta estimates, 95% confidence intervals and associated p-values are presented for each phenogroup, using phenogroup 3 as the baseline comparator. All analyses were adjusted for age, sex and ECG measurements (heart rate, QRS duration, QTc interval) and CHA_2DS_2-VASc score.

AF atrial fibrillation; BIDMC Beth Israel Deaconess Medical Centre; LA left atrial; LAVi left atrial volume indexed; LVEDD left ventricular end-diastolic diameter; LVEF left ventricular ejection fraction.

Supplementary Table 5

Phenogroup	CV death	All-cause mortality	Heart failure	Stroke	MACE
1	0.94 (0.75-1.16), 0.56	**0.88 (0.77-1.00), p < 0.05**	**1.25 (1.07-1.46), p < 0.01**	**1.41 (1.11-1.78), p < 0.01**	1.18 (1.00-1.39), 0.05
**2**	**1.27 (1.02-1.59), p < 0.05**	**1.17 (1.02-1.33), p < 0.05**	**1.60 (1.34-1.91), p < 0.0001**	**1.55 (1.20-2.00), p < 0.001**	**1.45 (1.20-1.75), p < 0.0001**
4	**0.73 (0.55-0.96), p < 0.05**	**0.71 (0.60-0.83), p < 0.0001**	1.01 (0.84-1.22), 0.88	1.16 (0.87-1.54), 0.33	1.00 (0.82-1.22), 0.99
5	**1.49 (1.17-1.89), p < 0.01**	**1.37 (1.18-1.58), p < 0.0001**	**1.78 (1.43-2.22), p < 0.0001**	1.20 (0.87-1.65), 0.27	**1.45 (1.13-1.87), p < 0.01**

Adjusted hazards ratio estimates from multivariate Cox proportional hazards models evaluating the associations between BIDMC AF phenogroups and incident cardiovascular disease and mortality outcomes. The adjusted hazards ratios (aHRs), 95% confidence intervals and associated p-values are presented for each phenogroup, using phenogroup 3 as the baseline comparator. All analyses were adjusted for age, sex and CHA_2DS_2-VASc score.

This analysis was performed as a sensitivity analysis using ECGs linked to echocardiography instances within 60 days.

AF atrial fibrillation; BIDMC Beth Israel Deaconess Medical Centre; CV cardiovascular; MACE major adverse cardiovascular events.

Supplementary Table 6

Phenogroup	CV death	All-cause mortality	Heart failure	Stroke	MACE
1	0.92 (0.71-1.18), 0.50	0.92 (0.79-1.08), 0.30	0.85 (0.70-1.03), 0.10	1.27 (0.95-1.68), 0.11	0.83 (0.67-1.03), 0.09
2	1.07 (0.83-1.38), 0.60	1.12 (0.95-1.30), 0.17	1.07 (0.87-1.33), 0.51	**1.56 (1.15-2.10), p < 0.01**	1.06 (0.84-1.33), 0.64
4	0.81 (0.60-1.10), 0.18	**0.78 (0.65-0.93), p < 0.01**	0.98 (0.80-1.21), 0.88	1.12 (0.81-1.54), 0.49	0.95 (0.76-1.18), 0.64
5	1.23 (0.94-1.62), 0.13	**1.28 (1.09-1.51), p < 0.01**	**1.46 (1.14-1.88), p < 0.01**	1.23 (0.86-1.76), 0.26	1.26 (0.94-1.70), 0.12

Adjusted hazards ratio estimates from multivariate Cox proportional hazards models evaluating the associations between BIDMC AF phenogroups and incident cardiovascular disease and mortality outcomes. The adjusted hazards ratios (aHRs), 95% confidence intervals and associated p-values are presented for each phenogroup, using phenogroup 3 as the baseline comparator. All analyses were adjusted for age, sex, CHA_2DS_2-VASc score and additionally LVEF %, LA size and AF/SR status at the time of the recording. This sensitivity analysis was performed using ECGs linked to echocardiography within 60 days to assess whether the derived phenogroups retained their associations beyond current clinical and echocardiographic variables.

AF atrial fibrillation; BIDMC Beth Israel Deaconess Medical Centre; CV cardiovascular; LA left atrial; LVEF left ventricular ejection fraction; MACE major adverse cardiovascular events.

Supplementary Table 7

Phenogroup	Correlation Coefficient with Sinus Rhythm
1	-0.32
2	-0.15
3	0.22
4	0.22
5	0.14

Correlation between phenogroup assignment and sinus rhythm status at the time of ECG acquisition in the BIDMC AF population.

AF atrial fibrillation; BIDMC Beth Israel Deaconess Medical Centre.

Supplementary Table 8

Variable	Phenogroup 1, N = 1,137[1]	Phenogroup 2, N = 252[1]	Phenogroup 3, N = 480[1]	Phenogroup 4, N = 446[1]	Phenogroup 5, N = 29[1]	p-value[2]
**Baseline variables**						
**Age**	71 (10)	72 (8)	71 (9)	71 (10)	74 (7)	0.011
**Females**	204 (18%)	50 (20%)	229 (48%)	208 (47%)	10 (34%)	<0.001
**CHA_2DS_2-VASc**	2 (2)	2 (2)	2 (2)	2 (2)	3 (2)	<0.001
**BMI**	26.8 (5.4)	27.6 (6.4)	26.9 (6.3)	26.8 (5.8)	30.1 (5.2)	<0.001
Unknown	25	3	14	9	1	
**Body Surface Area (m^2)**	1.97 (0.25)	2.00 (0.33)	1.92 (0.28)	1.87 (0.29)	1.96 (0.39)	<0.001
Unknown	682	150	283	271	20	
**Systolic Blood Pressure (mmHg)**	143 (26)	141 (26)	145 (28)	142 (27)	142 (24)	0.15
Unknown	222	50	105	95	3	
**Diastolic Blood Pressure (mmHg)**	80 (17)	80 (17)	77 (15)	78 (14)	79 (9)	0.001
Unknown	222	50	105	95	3	
**ECG measures**						
**Heart Rate**	61 (19)	68 (17)	62 (15)	58 (11)	64 (22)	<0.001
Unknown	433	88	171	174	13	
**QRS interval**	88 (16)	92 (24)	90 (18)	90 (22)	122 (52)	<0.001
**QTc interval**	422 (35)	434 (43)	431 (31)	428 (29)	452 (31)	<0.001
Unknown	433	88	171	174	13	
**PR interval**	178 (48)	184 (42)	168 (31)	164 (28)	160 (14)	<0.001
Unknown	785	183	217	199	16	
**Sinus rhythm**	605 (53%)	124 (49%)	437 (91%)	415 (93%)	23 (79%)	<0.001
**Cardiac MRI measures**						
**LVEF (%)**	56 (11)	51 (14)	60 (10)	60 (8)	61 (16)	<0.001
Unknown	697	156	293	274	19	
**LVEDV (ml)**	161 (46)	156 (57)	150 (51)	151 (52)	154 (85)	0.018
Unknown	697	156	293	274	19	
**LAEF (%)**	48 (34)	34 (33)	55 (15)	56 (14)	47 (22)	<0.001
Unknown	706	158	297	277	19	
**LAV min indexed (ml/m^2)**	26 (26)	33 (30)	19 (15)	19 (12)	26 (42)	<0.001
Unknown	713	160	302	283	20	

**LAV max indexed (ml/m^2)**	50 (24)	54 (23)	42 (19)	44 (16)	44 (49)	<0.001
Unknown	713	160	302	283	20	
**LASV indexed (ml/m^2)**	21 (11)	19 (9)	22 (10)	23 (8)	21 (10)	<0.001
Unknown	713	160	302	283	20	
**Circumferential PDSR**	1.92 (1.18)	1.55 (0.79)	2.08 (1.19)	2.44 (1.05)	1.99 (0.75)	<0.001
Unknown	610	139	256	235	18	
**Longitudinal PDSR**	1.54 (0.88)	1.33 (0.67)	1.53 (0.73)	1.68 (0.81)	1.32 (0.90)	<0.001
Unknown	618	140	259	238	17	
**Radial PDSR**	-5.13 (2.94)	-4.18 (1.98)	-5.42 (3.18)	-6.10 (2.31)	-5.65 (1.68)	<0.001
Unknown	611	139	256	235	18	
**Smoking**						
Current	30 (2.7%)	5 (2.0%)	13 (2.7%)	8 (1.8%)	2 (6.9%)	
Never	618 (55%)	134 (54%)	275 (58%)	248 (56%)	6 (21%)	
Prefer not to answer	3 (0.3%)	2 (0.8%)	1 (0.2%)	4 (0.9%)	0 (0%)	
Previous	472 (42%)	109 (44%)	189 (40%)	180 (41%)	21 (72%)	
Unknown	14	2	2	6	0	
**Prevalent disease**						
**Hypertension**	642 (56%)	166 (66%)	287 (60%)	210 (47%)	25 (86%)	<0.001
**Stroke**	59 (5.2%)	12 (4.8%)	21 (4.4%)	20 (4.5%)	2 (6.9%)	0.88
**Diabetes Mellitus**	119 (10%)	38 (15%)	70 (15%)	30 (6.7%)	7 (24%)	
**Chronic Kidney Disease**	63 (5.5%)	28 (11%)	25 (5.2%)	19 (4.3%)	6 (21%)	<0.001
**Myocardial Infarction**	90 (7.9%)	33 (13%)	56 (12%)	30 (6.7%)	7 (24%)	<0.001
**COPD**	28 (2.5%)	13 (5.2%)	18 (3.8%)	20 (4.5%)	5 (17%)	0.001
**Heart failure**	82 (7.2%)	49 (19%)	49 (10%)	23 (5.2%)	7 (24%)	
**Mortality outcomes**						
**All-cause mortality**	26 (2.3%)	10 (4.0%)	10 (2.1%)	9 (2.0%)	0 (0%)	0.51
[1] Median (IQR); n (%)						
[2] Kruskal-Wallis rank sum test; Pearson's Chi-squared test; Fisher's exact test						

Descriptive statistics of the UKB AF DDRTree phenogroups across baseline variables, cardiac MRI measures and disease outcomes. Continuous variables are shown as median values with the associated interquartile ranges and categorical variables are shown with their frequencies. Differences across phenogroups were quantified using the non-

parametric Kruskal-Wallis rank sum test and Pearson’s Chi-squared test for continuous and categorical variables, respectively using a p-value significance threshold of 0.05. Unknown values refer to missing values for variables.

AF atrial fibrillation; BMI body mass index; COPD chronic obstructive pulmonary disease; MRI magnetic resonance imaging; DDRTree dimensionality reduction via learning a tree; LA left atrial; LAEF left atrial ejection fraction; LASV left atrial systolic volume; LAV left atrial volume; LVEDD left ventricular end-diastolic diameter; LVEDV left ventricular end-diastolic volume; LVEF left ventricular ejection fraction; PDSR peak diastolic strain rate; UKB UK Biobank.

Supplementary Table 9

Phenogroup	Hypertension	Diabetes mellitus	Chronic kidney disease	Myocardial infarction	Heart failure	Stroke
1	0.80 (0.64 - 1.01), 0.06	**0.61 (0.44 - 0.84), p < 0.01**	1.09 (0.67 - 1.81), 0.74	**0.47 (0.33 - 0.68), p < 0.0001**	**0.60 (0.41 - 0.88), p < 0.01**	1.15 (0.69 - 1.99), 0.60
2	1.14 (0.82 - 1.59), 0.44	0.91 (0.58 - 1.40), 0.66	**2.21 (1.24 - 3.95), p < 0.01**	0.80 (0.49 -1.28), 0.36	**1.81 (1.16 - 2.81), p < 0.01**	1.00 (0.46 - 2.05), 0.99
4	**0.61 (0.46 - 0.79), p < 0.001**	**0.42 (0.27 - 0.66), p < 0.001**	0.85 (0.46 - 1.57), 0.61	**0.54 (0.33 - 0.86), p < 0.05**	**0.49 (0.29 - 0.81), p < 0.01**	1.07 (0.57 - 2.02), 0.82
5	**3.89 (1.46 - 13.47), p < 0.05**	1.72 (0.66 - 4.02), 0.23	**4.56 (1.56 - 11.78), p < 0.01**	2.02 (0.75 -4.90), 0.14	**2.52 (0.95 - 6.02), p < 0.05**	1.49 (0.23 - 5.49), 0.61

Adjusted odds ratio estimates from multivariate logistic models evaluating the associations between UKB AF phenogroups and prevalent disease outcomes. The adjusted odds ratios (aORs), 95% confidence intervals and associated p-values are presented for each phenogroup, using phenogroup 3 as the baseline comparator. All analyses were adjusted for age and sex.

AF atrial fibrillation; UKB UK Biobank.

Supplementary Table 10

Phenogroup	MACE	Heart Failure	Stroke	All-cause mortality
1	0.87 (0.41- 1.84), 0.71	0.70 (0.37-1.34), 0.28	**4.78 (1.09-20.88), p < 0.05**	1.05 (0.50-2.23), 0.89
2	1.72 (0.70- 4.22), 0.23	1.79 (0.85-3.77), 0.13	1.93 (0.27-13.91), 0.52	1.69 (0.69-4.13), 0.25
4	1.38 (0.61- 3.11), 0.44	0.39 (0.14-1.07), 0.07	3.52 (0.71-17.52), 0.12	1.07 (0.43-2.63), 0.89
5	2.09 (0.27-16.40), 0.48	0.97 (0.13-7.42), 0.98	0.00 (0.00- Inf), 1.00	0.00 (0.00- Inf), 0.99

Adjusted hazards ratio estimates from multivariate Cox proportional hazards models evaluating the associations between UKB AF phenogroups and incident cardiovascular disease and mortality outcomes. The adjusted hazards ratios (aHRs), 95% confidence intervals and associated p-values are presented for each phenogroup, using phenogroup 3 as the baseline comparator. All analyses were adjusted for age and sex.

AF atrial fibrillation; MACE major adverse cardiovascular events; UKB UK Biobank.

Supplementary Table 11

Pheno group	LVEF (%)	LVEDV (ml)	LAEF (%)	LAV min indexed (ml/m^2)	LAV max indexed (ml/m^2)	LASV indexed (ml/m^2)	Circumferential PDSR	Longitudinal PDSR	Radial PDSR
1	**-1.85 (-3.27 to -0.44), p < 0.05**	-4.16 (-9.84 to 1.52), 0.15	**-9.73 (-12.45 to -7.01), p < 0.0001**	**10.56 (7.47 to 13.65), p < 0.0001**	**9.50 (6.22 to 12.78), p < 0.0001**	-1.06 (-2.38 to 0.26), 0.12	-0.02 (-0.14 to 0.11), 0.79	0.06 (-0.05 to 0.16), 0.29	0.01 (-0.30 to 0.32), 0.96
2	**-5.81 (-7.82 to -3.80), p < 0.0001**	-5.94 (-14.02 to 2.14), 0.15	**-13.10 (-16.97 to -9.23), p < 0.0001**	**11.56 (7.16 to 15.96), p < 0.0001**	**9.44 (4.77 to 14.11), p < 0.0001**	**-2.12 (-4.00 to -0.24), p < 0.05**	**-0.37 (-0.55 to -0.20), p < 0.0001**	-0.13 (-0.28 to 0.02), 0.09	**0.93 (0.48 to 1.38), p < 0.0001**
4	0.52 (-1.16 to 2.20), 0.54	-0.22 (-6.95 to 6.51), 0.95	-1.40 (-4.62 to 1.82), 0.40	0.96 (-2.72 to 4.63), 0.61	1.74 (-2.17 to 5.64), 0.38	0.78 (-0.79 to 2.35), 0.33	**0.21 (0.06 to 0.36), p < 0.01**	**0.15 (0.02 to 0.27), p < 0.05**	**-0.47 (-0.84 to -0.11), p < 0.05**
5	-0.02 (-5.14 to 5.11), 0.99	0.32 (-20.25 to 20.89), 0.98	-6.43 (-16.18 to 3.32), 0.20	10.01 (-1.51 to 21.54), 0.09	9.77 (-2.46 to 22.00), 0.12	-0.24 (-5.17 to 4.69), 0.92	-0.14 (-0.61 to 0.33), 0.57	-0.20 (-0.57 to 0.17), 0.30	0.01 (-1.17 to 1.19), 0.99

Adjusted beta estimates from multivariate linear models evaluating the associations between UKB AF phenogroups and cardiac MRI measures of LV/LA structure and function. The adjusted beta estimates, 95% confidence intervals and associated p-values are presented for each phenogroup, using phenogroup 3 as the baseline comparator. All analyses were adjusted for age and sex. *AF atrial fibrillation; LAEF left atrial emptying fraction; LAV left atrial volume; LASV left atrial systolic volume; LVEDV left ventricular end-diastolic volume; LVEF left ventricular ejection fraction; MRI magnetic resonance imaging; PDSR peak diastolic strain rate; UKB UK Biobank.*

Supplementary Table 12

	Hypertension	Diabetes	Obesity	Mitral Regurgitation	Chronic Kidney Disease	Myocardial Infarction	Heart Failure	Stroke	Peripheral Vascular Disease
Age	1.037	1.041	1.048	1.044	1.039	1.044	1.030	1.043	1.044
Female	1.099	1.100	1.099	1.102	1.099	1.093	1.097	1.105	1.103
QRS	1.299	1.332	1.334	1.324	1.360	1.359	1.300	1.341	1.359
Heart Rate	1.142	1.132	1.140	1.135	1.123	1.115	1.132	1.123	1.121
QTc	1.243	1.248	1.254	1.250	1.256	1.273	1.214	1.249	1.256
Phenogroup assignment	1.054	1.056	1.058	1.056	1.056	1.056	1.050	1.057	1.058

Generalised variance inflation factor (GVIF) values across covariates included in multivariate logistic models for the BIDMC AF population. *AF atrial fibrillation; BIDMC Beth Israel Deaconess Medical Centre.*

Supplementary Figure 1

a

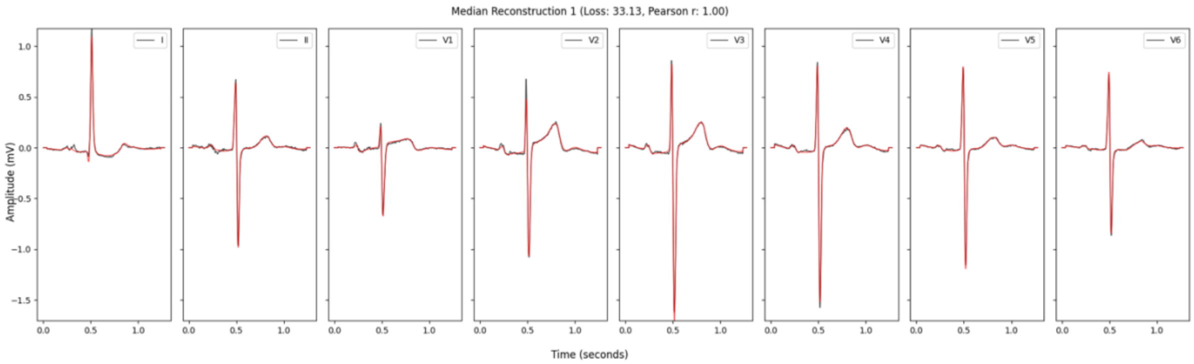

b

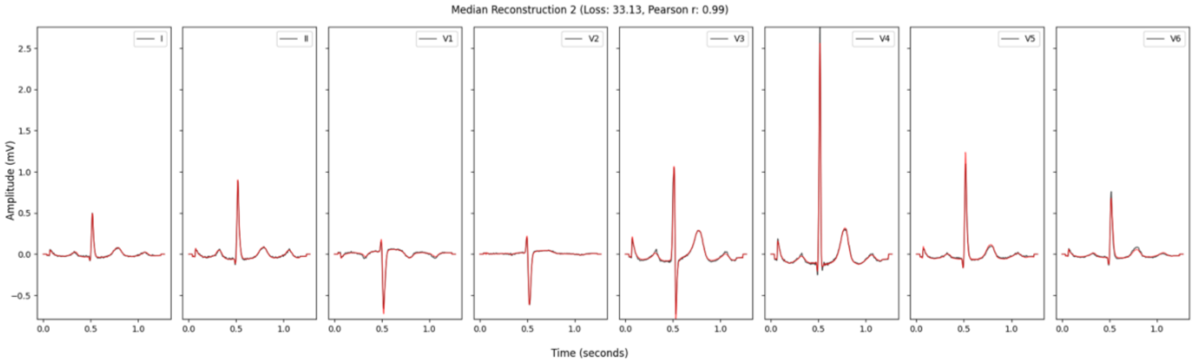

c

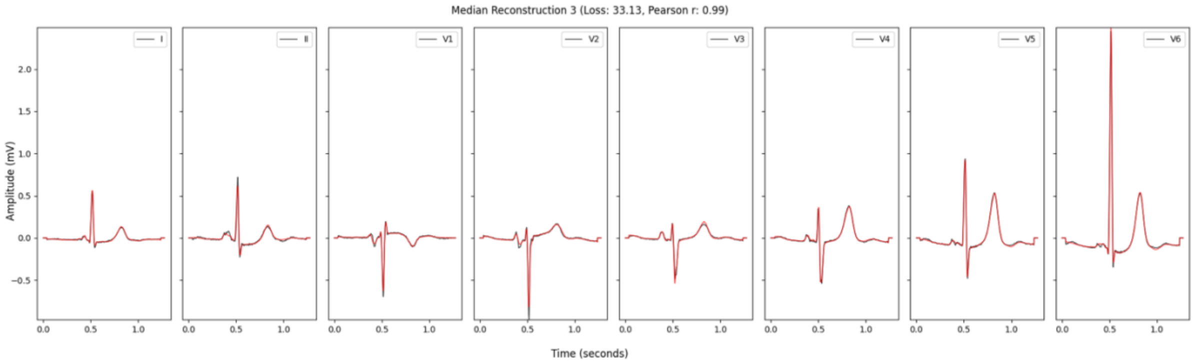

d

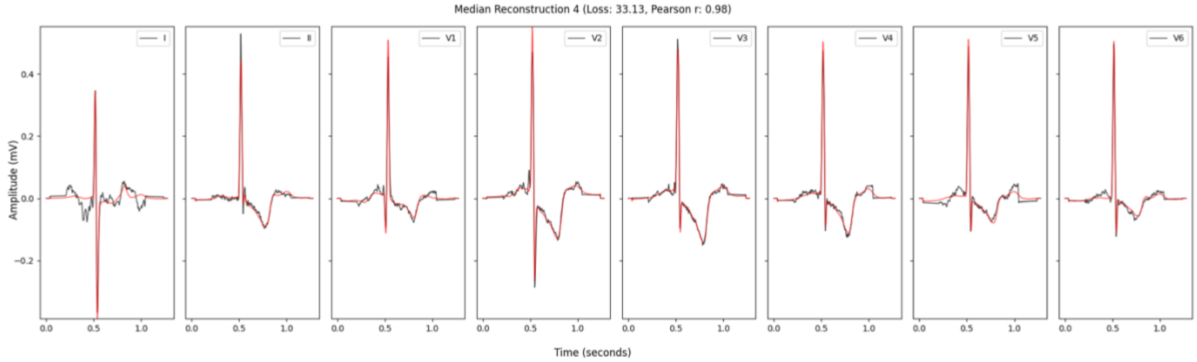

e

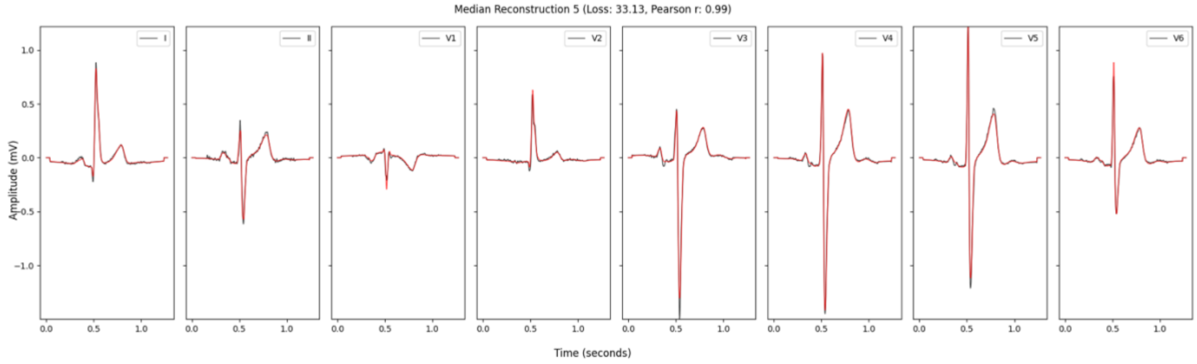

f

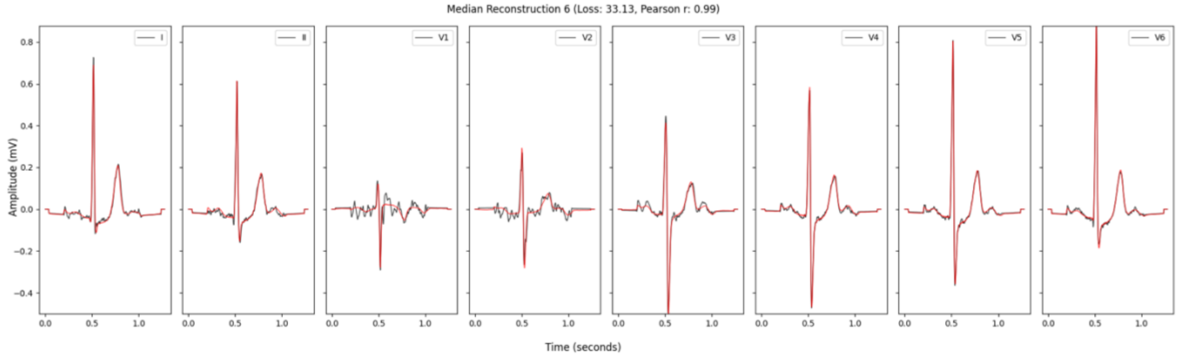

Examples of median beat reconstruction from the BIDMC AF population using the trained variational autoencoder model. Subplots a-f show reconstructed 8-lead median beat signals for a random subset of patients from the BIDMC AF subset. The

black line indicates the original signal while the red line indicates the reconstructed signal from the model.

AF atrial fibrillation; BIDMC Beth Israel Deaconess Medical Centre.

Supplementary Figure 2

a

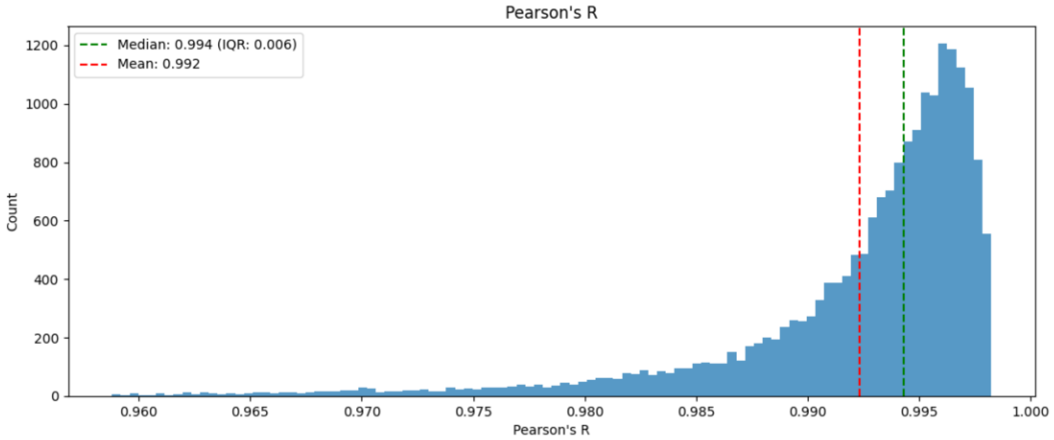

b

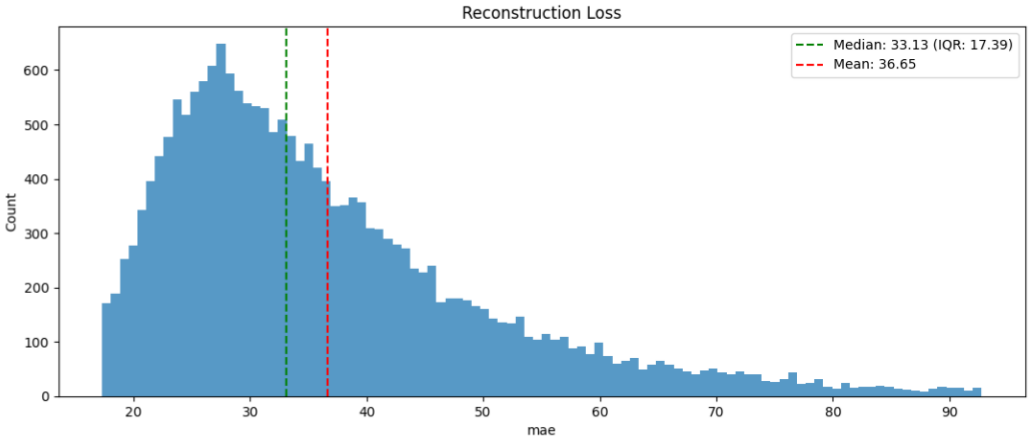

Performance of the variational autoencoder for median beat reconstruction in the AF subset of the BIDMC cohort. a) Pearson's R and b) Reconstruction loss for the BIDMC cohort.

AF atrial fibrillation, BIDMC Beth Israel Deaconess Medical Centre.

Supplementary Figure 3

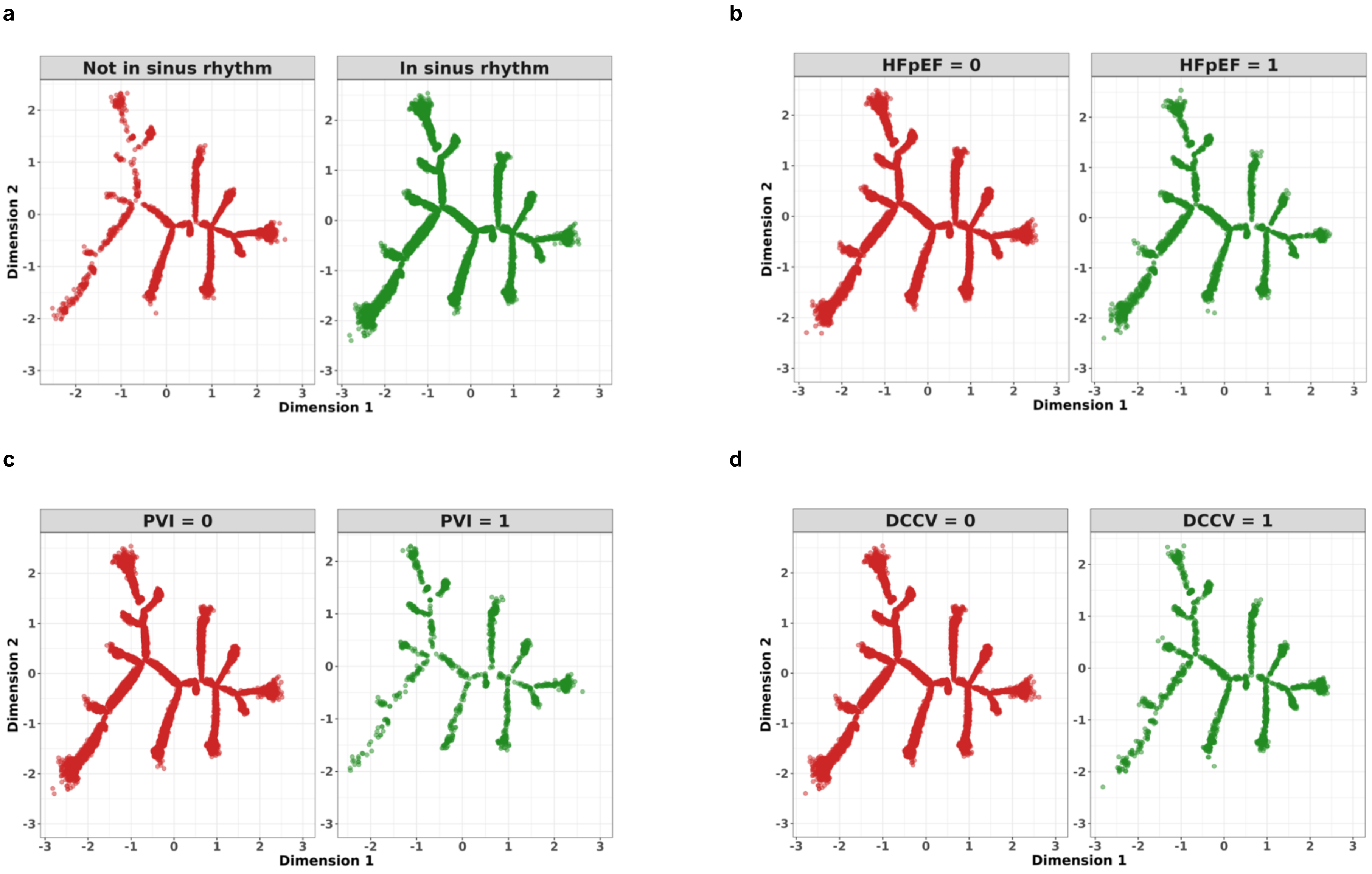

Overlaying AF-related phenotypes across the BIDMC AF DDRTree. Subplot a) shows the proportion of ECGs recorded in sinus rhythm. Subplot b) shows the presence of HFpEF. Subplot c) shows the distribution of pulmonary-vein isolation (PVI) therapy during follow-up. Subplot d) shows the distribution of direct-current cardioversion (DCCV) therapy during follow-up.

AF atrial fibrillation; BIDMC Beth Israel Deaconess Medical Centre; DDRTree dimensionality reduction via learning a tree; DCCV direct current cardioversion; HFpEF heart failure with preserved ejection fraction; PVI pulmonary vein isolation.

Supplementary Figure 4

a

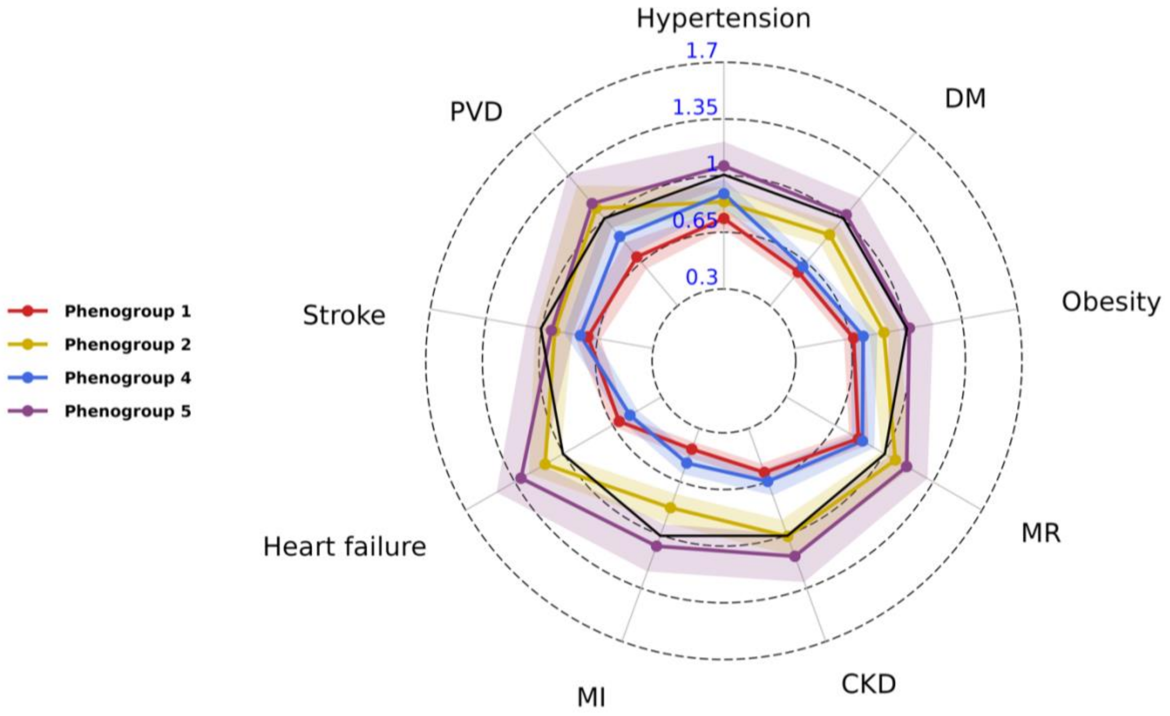

b

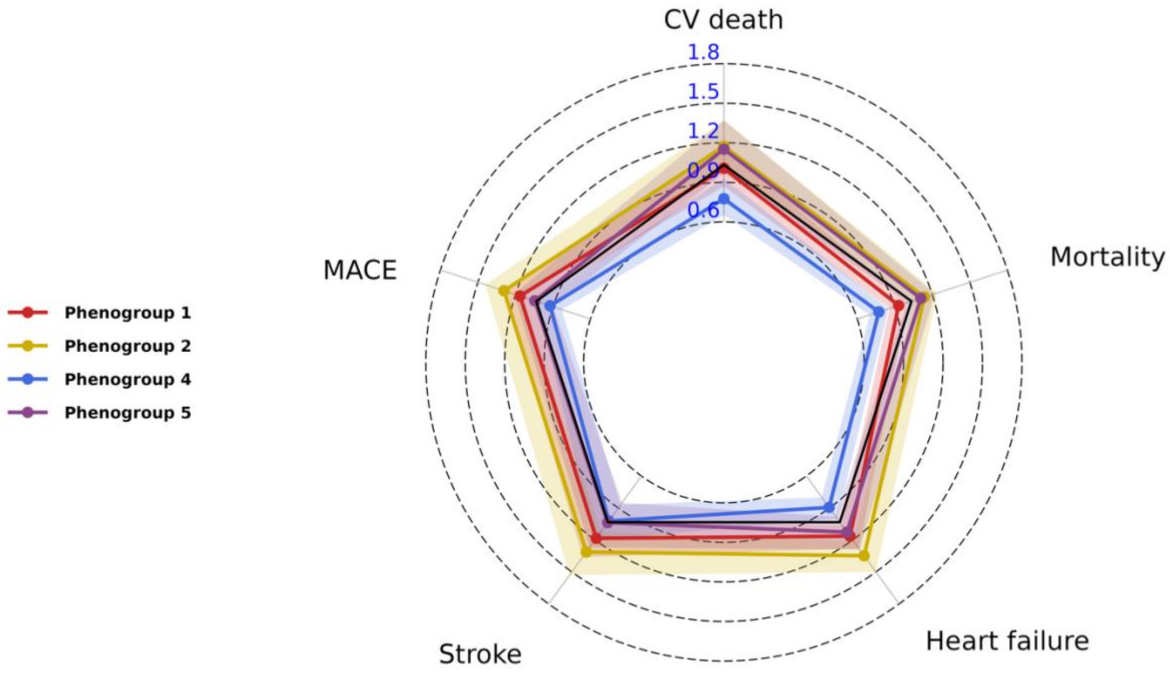

Radar plots showing the associations of BIDMC AF DDRTree phenogroups against cardiovascular comorbidities and mortality outcomes. Subplot a) shows the adjusted odds ratios and 95% confidence intervals for each phenogroup against prevalent diseases, using phenogroup 3 as the baseline comparator. Subplot b) shows the adjusted hazards ratios and 95% confidence intervals for each phenogroup against incident diseases, using phenogroup 3 as the baseline comparator. All models presented here were adjusted for age, sex, heart rate, QRS duration, QTc interval while incident disease models were additionally adjusted for CHA_2DS_2-VASc score.

AF atrial fibrillation; BIDMC Beth Israel Deaconess Medical Centre; CKD chronic kidney disease; CV cardiovascular DM diabetes mellitus; DDRTree dimensionality reduction via learning a tree; MACE major adverse cardiovascular events; MR mitral regurgitation; MI myocardial infarction; PVD peripheral vascular disease.

Supplementary Figure 5

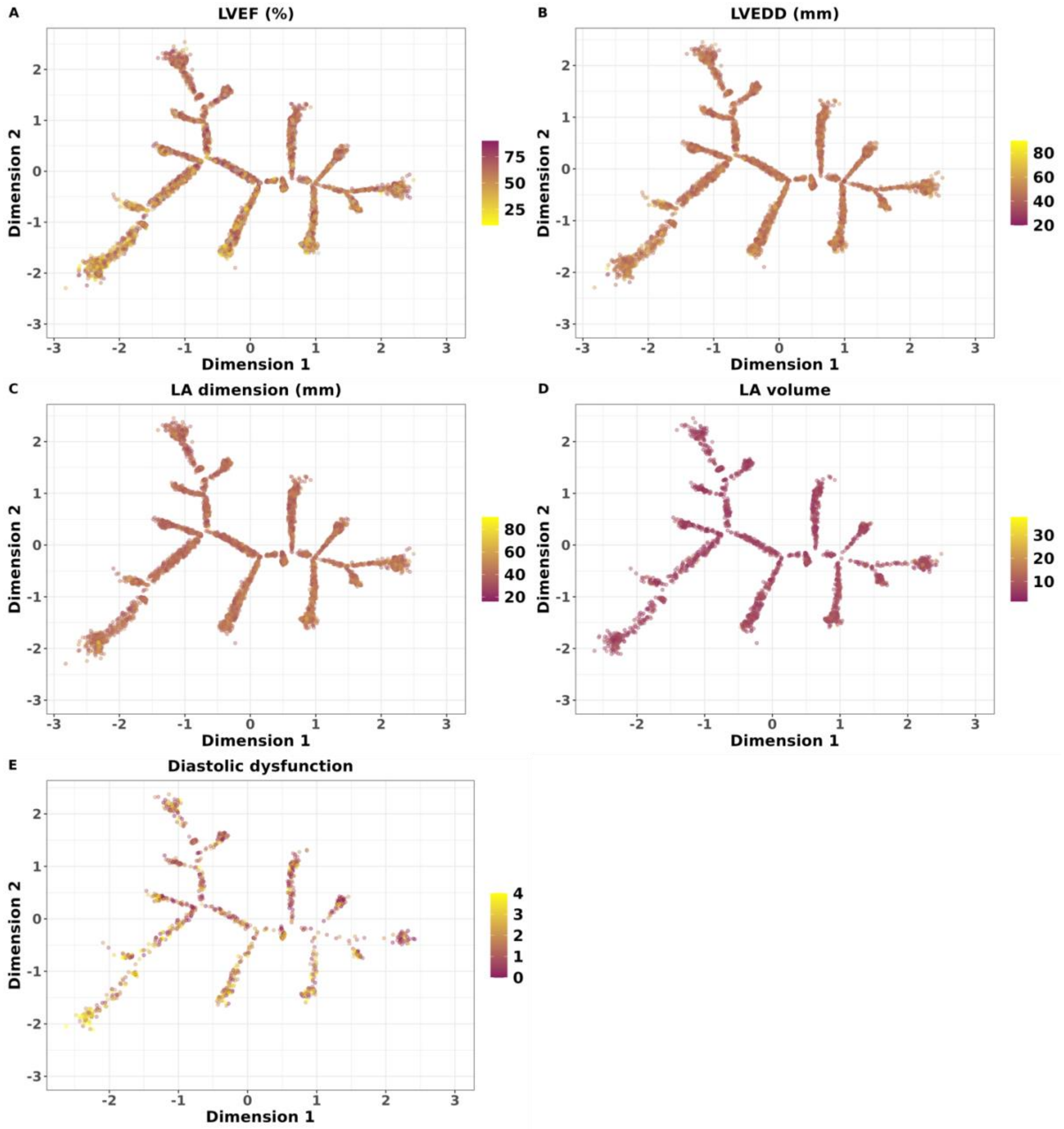

Overlaying echocardiography measures of left ventricular and atrial structure and function across the BIDMC AF DDRTree.

AF atrial fibrillation; BIDMC Beth Israel Deaconess Medical Centre; DDRTree dimensionality reduction via learning a tree; LA left atrial; LVEDD left ventricular end-diastolic diameter; LVEF left ventricular ejection fraction.

Supplementary Figure 6

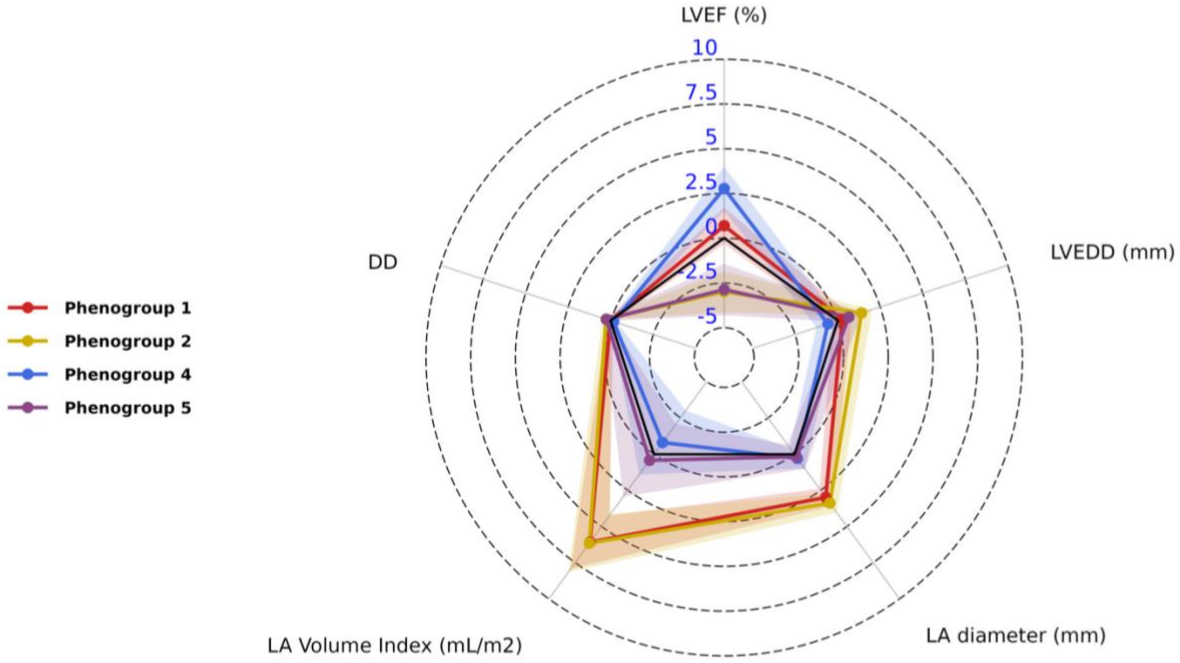

Radar plot showing the associations of BIDMC AF DDRTree phenogroups against echocardiography measures of LV/LA function and size. The plot shows the adjusted beta estimates and 95% confidence intervals for each phenogroup against different measures, using phenogroup 3 as the baseline comparator. All models presented here were adjusted for age, sex, heart rate, QRS duration, QTc interval and CHA_2DS_2-VASc score.

AF atrial fibrillation; BIDMC Beth Israel Deaconess Medical Centre; DD diastolic dysfunction; DDRTree dimensionality reduction via learning a tree; DD diastolic dysfunction; LA left atrial; LVEDD left ventricular end-diastolic diameter; LVEF left ventricular ejection fraction.

Supplementary Figure 7

a

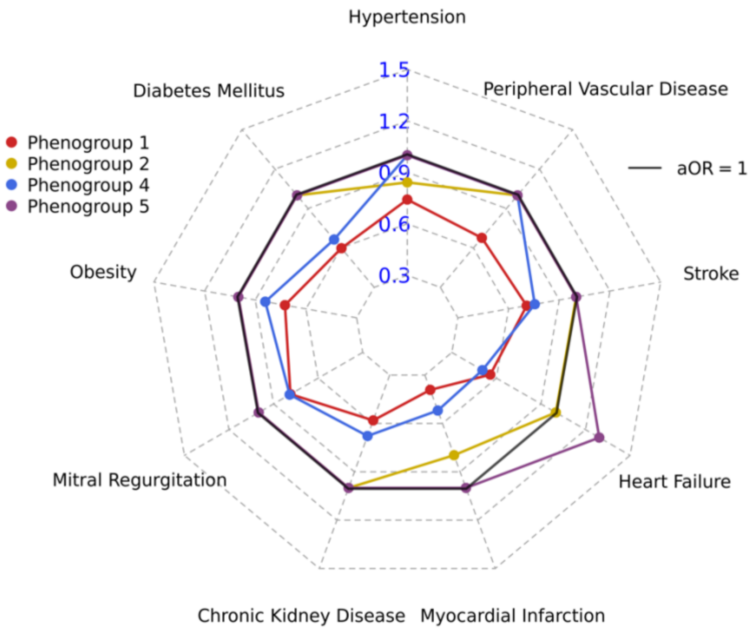

b

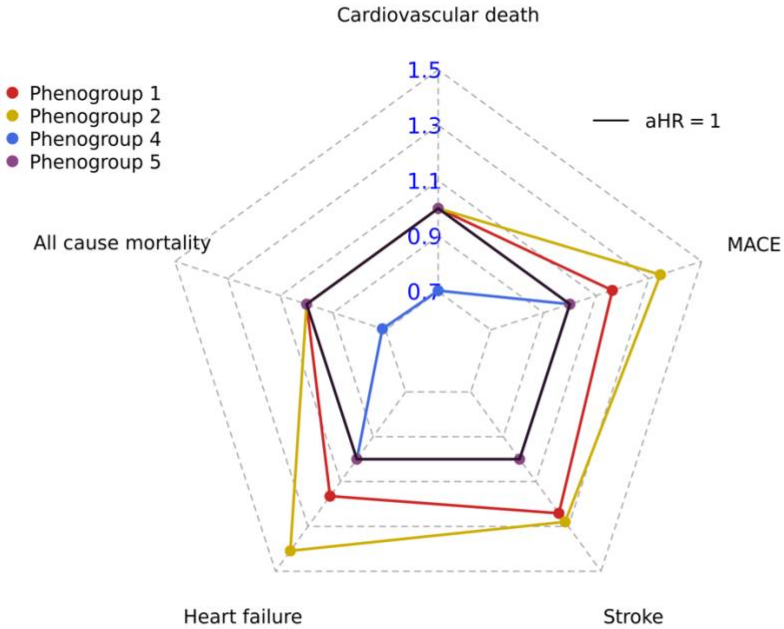

c

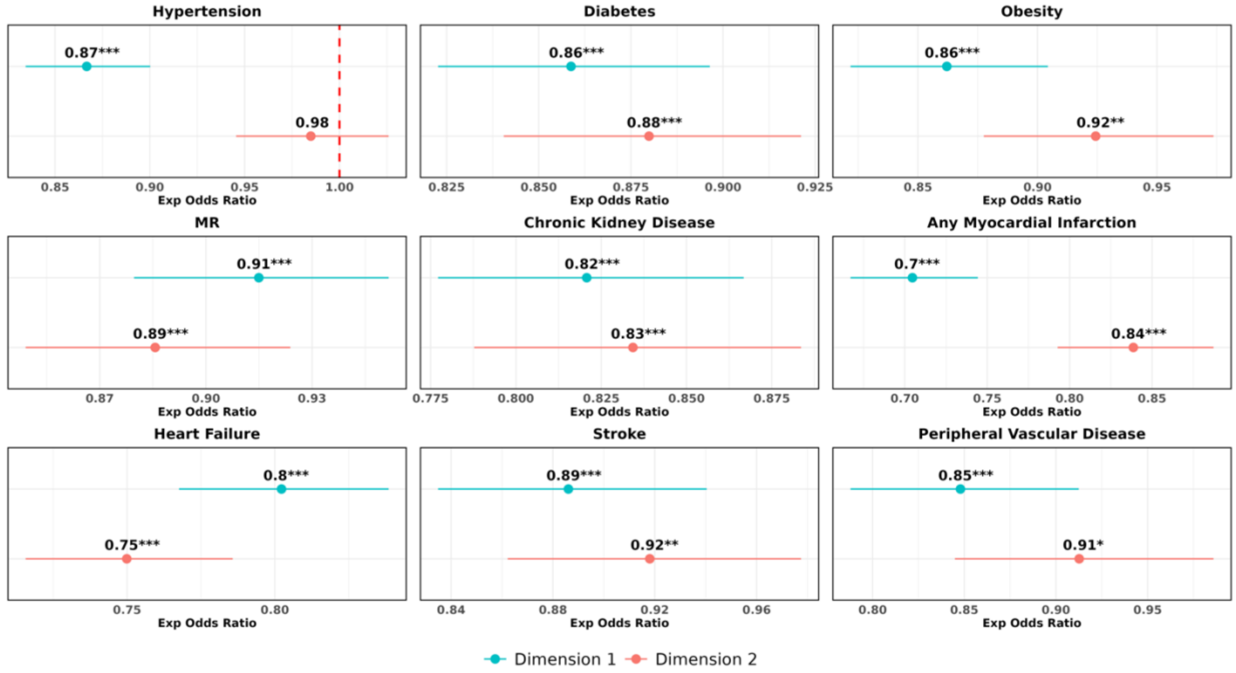

d

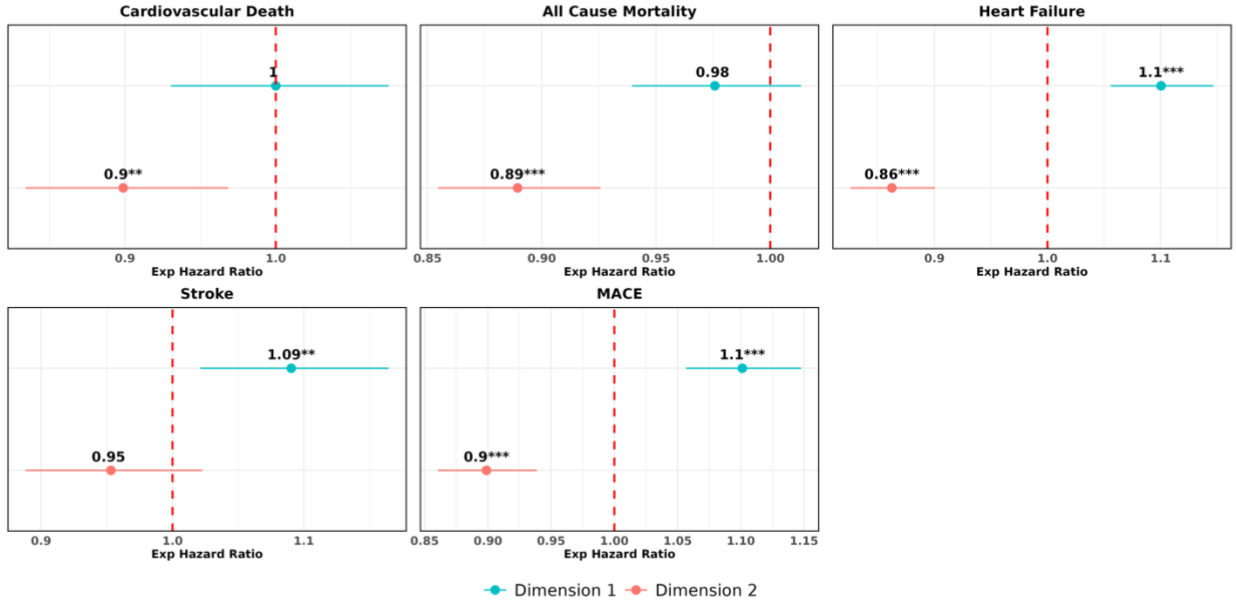

Sensitivity analysis restricting disease analyses to subsequent ECG taken within 60 days after the AF diagnosis. Subplot a) is a radar plot showing the adjusted odds ratios (aOR) for the different phenogroups against prevalent disease, using phenogroup

3 as the baseline comparator. Regression estimates that did not reach statistical significance (p-value > 0.05) were set at an aOR of 1 in the plot. Subplot b) is a radar plot showing the adjusted hazards ratios (aHR) for the different phenogroups against incident disease, using phenogroup 3 as the baseline comparator. Regression estimates that did not reach statistical significance (p-value > 0.05) were set at an aHR of 1 in the plot. Panel C) contains forest plots showing the aORs of the tree dimensions against prevalent diseases, adjusted for covariates. Panel D) contains forest plots showing the aHRs of the tree dimensions against incident diseases, adjusted for covariates. The horizontal red lines indicate an aOR/aHR of 1, indicating non-significance. All regression models presented were adjusted for age, sex, heart rate, QRS duration, QTc interval while incident disease models were additionally adjusted for CHA_2DS_2-VASc score.

AF atrial fibrillation; MACE major adverse cardiovascular events; MR mitral regurgitation.

Supplementary Figure 8

a

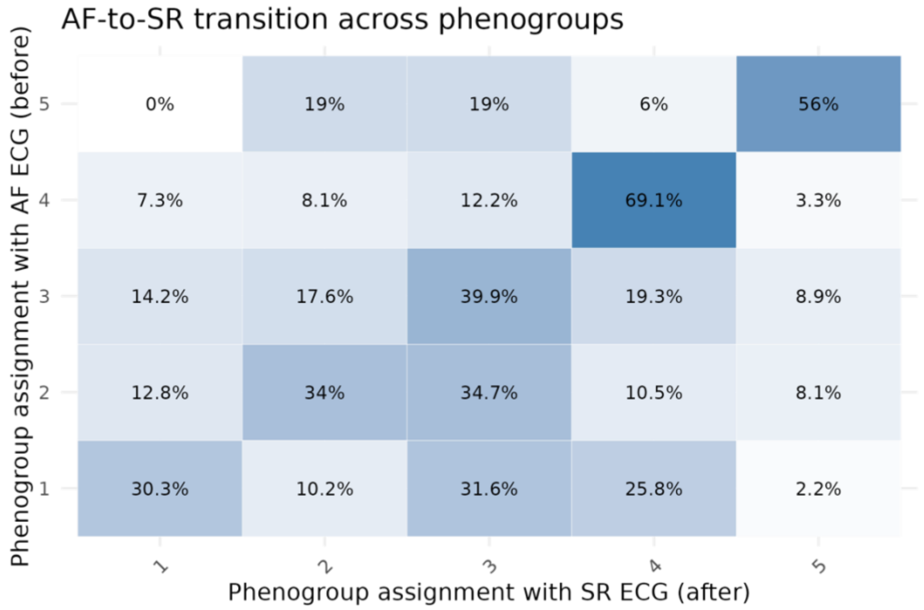

b

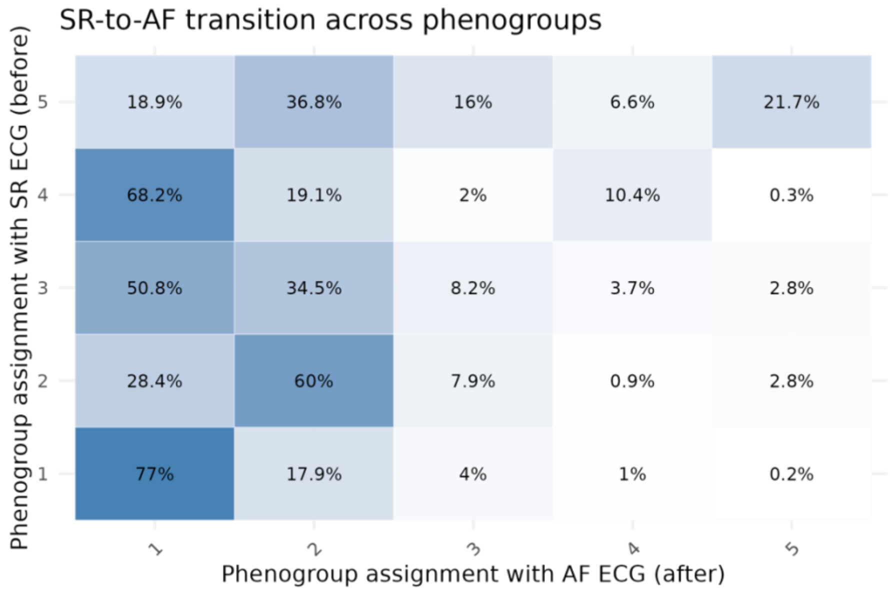

Sensitivity analyses investigating the effect of rhythm on phenogroup assignment stability in the AF BIDMC cohort. Subplot a) shows the transition matrix

with values (percentages) representing transitions from AF on ECG (y-axis) to SR on ECG (x-axis) and the corresponding changes in phenogroup assignment. Subplot b) shows the transition matrix with values (percentages) representing transitions from SR on ECG (y-axis) to AF on ECG (x-axis) and the corresponding changes in phenogroup assignment. All ECG pairs were within a 1-month timeframe.

AF atrial fibrillation; BIDMC Beth Israel Deaconess Medical Centre; SR sinus rhythm.

Supplementary Figure 9

a

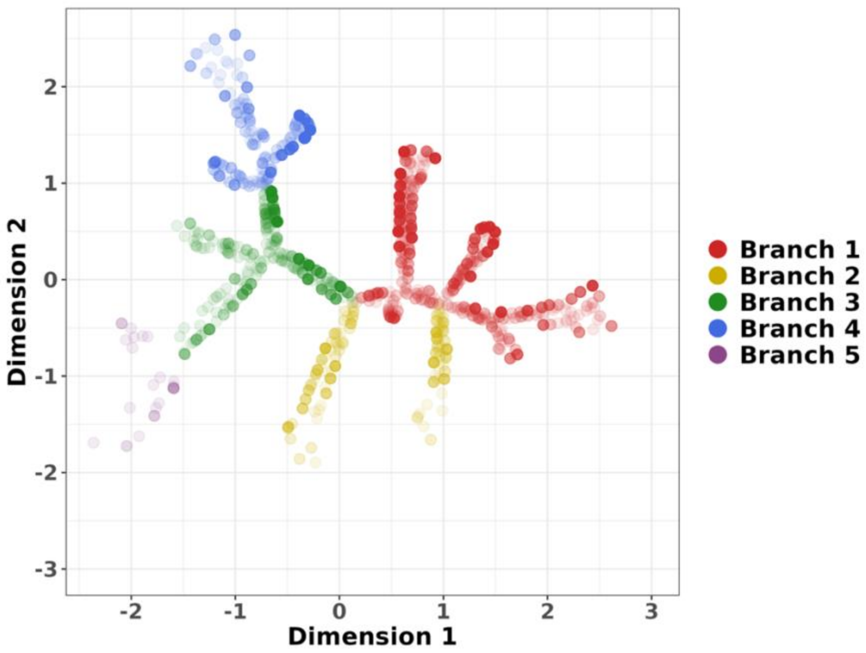

b

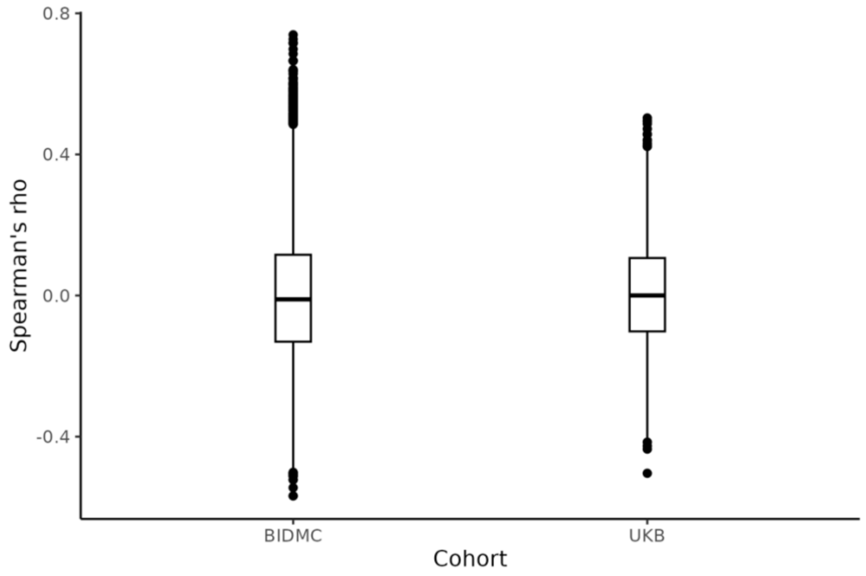

Projecting the UKB AF population onto the BIDMC AF DDRTree for external validation. Subplot a) shows the predicted tree variables (colored branch assignments and dimension coordinates) for the UKB cohort predicted using supervised models

trained on BIDMC AF DDRTree data. Subplot b) shows the similarity between nearest tree points across the BIDMC and UKB AF cohorts. The plot indicates that the Spearman's correlation coefficients between the latent features of the nearest points of BIDMC points in the tree follow the same distribution as the nearest UKB and BIDMC points.

AF atrial fibrillation; BIDMC Beth Israel Deaconess Medical Centre; DDRTree dimensionality reduction via learning a tree; UKB UK Biobank.

Supplementary Figure 10

a

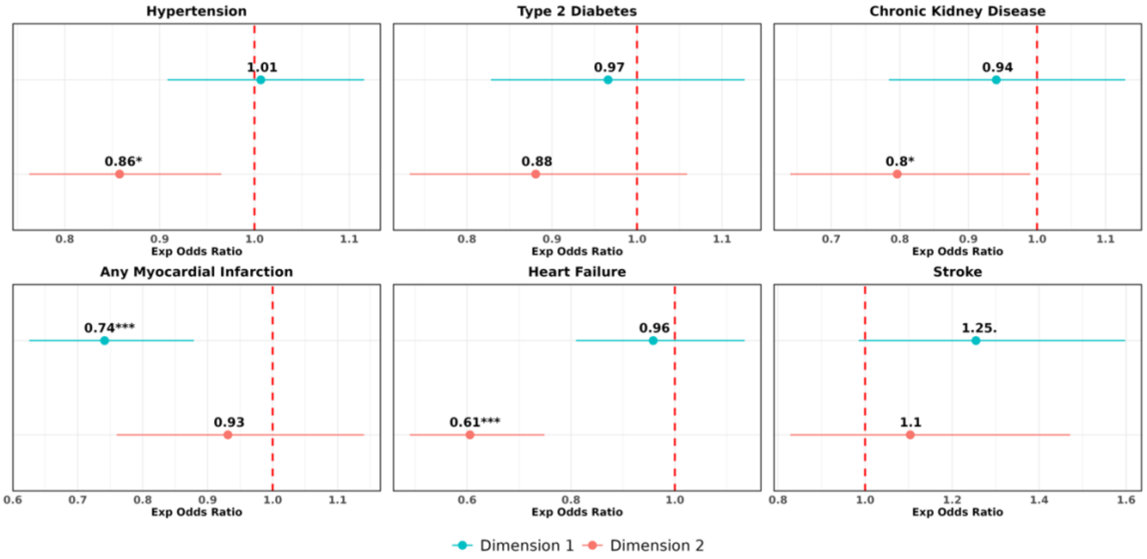

b

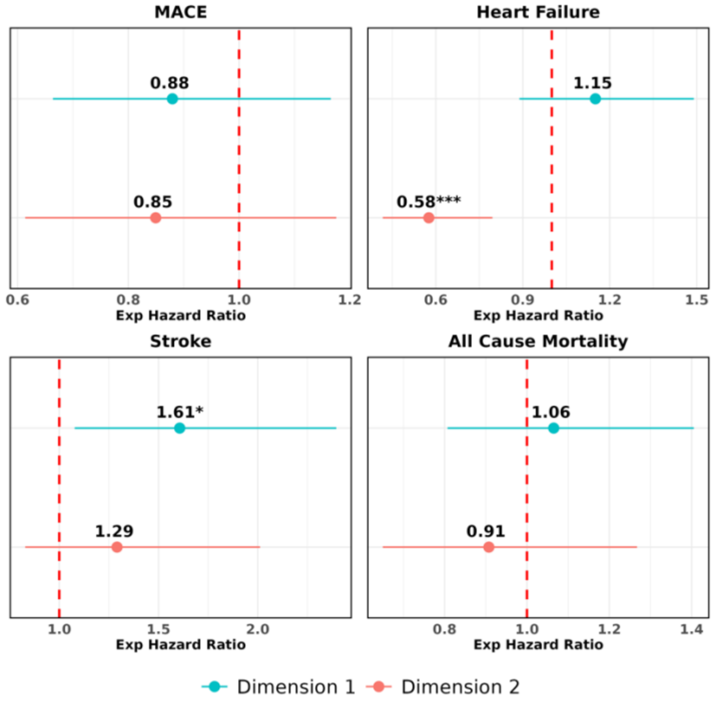

Multivariate regressions of the UKB AF tree dimensions (1 and 2) against prevalent and incident disease outcomes. Panel A) contains forest plots showing the

adjusted odds ratios (aOR) of tree dimensions against prevalent disease, adjusted for covariates. Panel B) contains forest plots showing the adjusted hazards ratios (aHR) of tree dimensions against incident disease outcomes, adjusted for covariates. The horizontal red lines indicate an aOR/aHR of 1, indicating non-significance. All models were adjusted for age, sex while the incident disease models were additionally adjusted for CHA_2DS_2-VASc score.

AF atrial fibrillation; UKB UK Biobank; MACE major adverse cardiovascular events.

Supplementary Figure 11

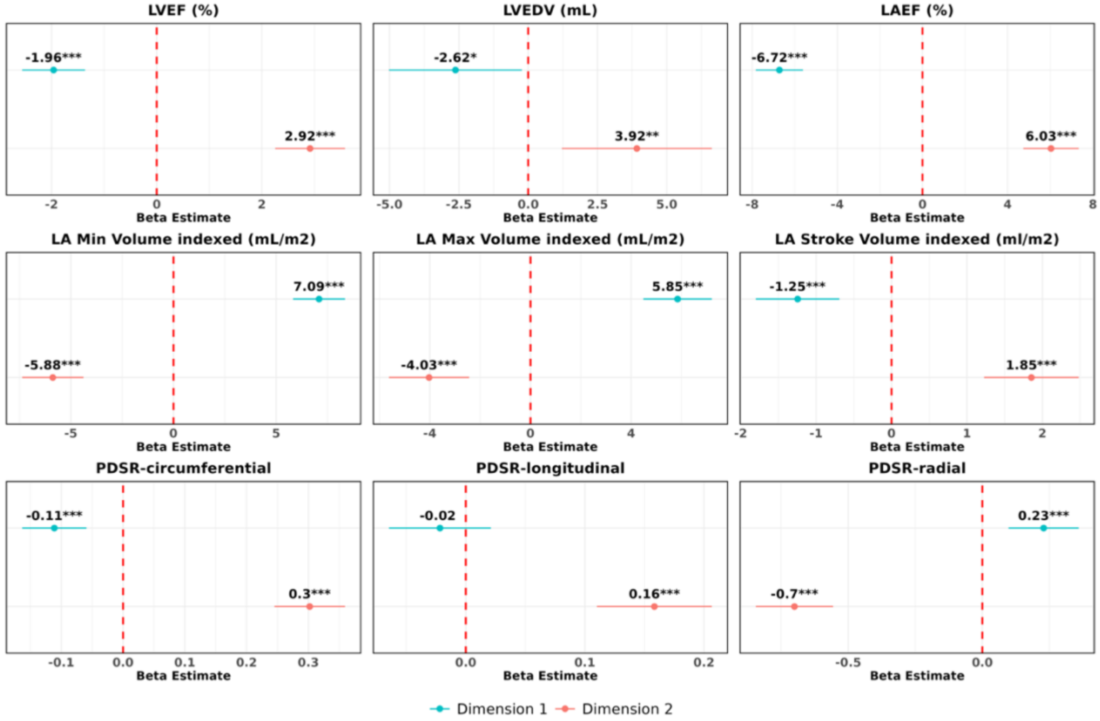

Multivariate regressions of the UKB tree dimensions (1 and 2) against cardiac MRI measures of left atrial/ventricular function and structure. The panel consists of forest plots showing the adjusted beta estimates of tree dimensions against various measures, adjusted for covariates. The horizontal red lines indicate an adjusted beta estimates of 0, indicating non-significance. All models were adjusted for age, sex and CHA_2DS_2-VASc score.

AF atrial fibrillation; LAEF left atrial emptying fraction; LAV left atrial volume; LASV left atrial systolic volume; LVEDV left ventricular end-diastolic volume; LVEF left ventricular ejection fraction; MRI magnetic resonance imaging; PDSR peak diastolic strain rate; UKB UK Biobank.

Supplementary Figure 12

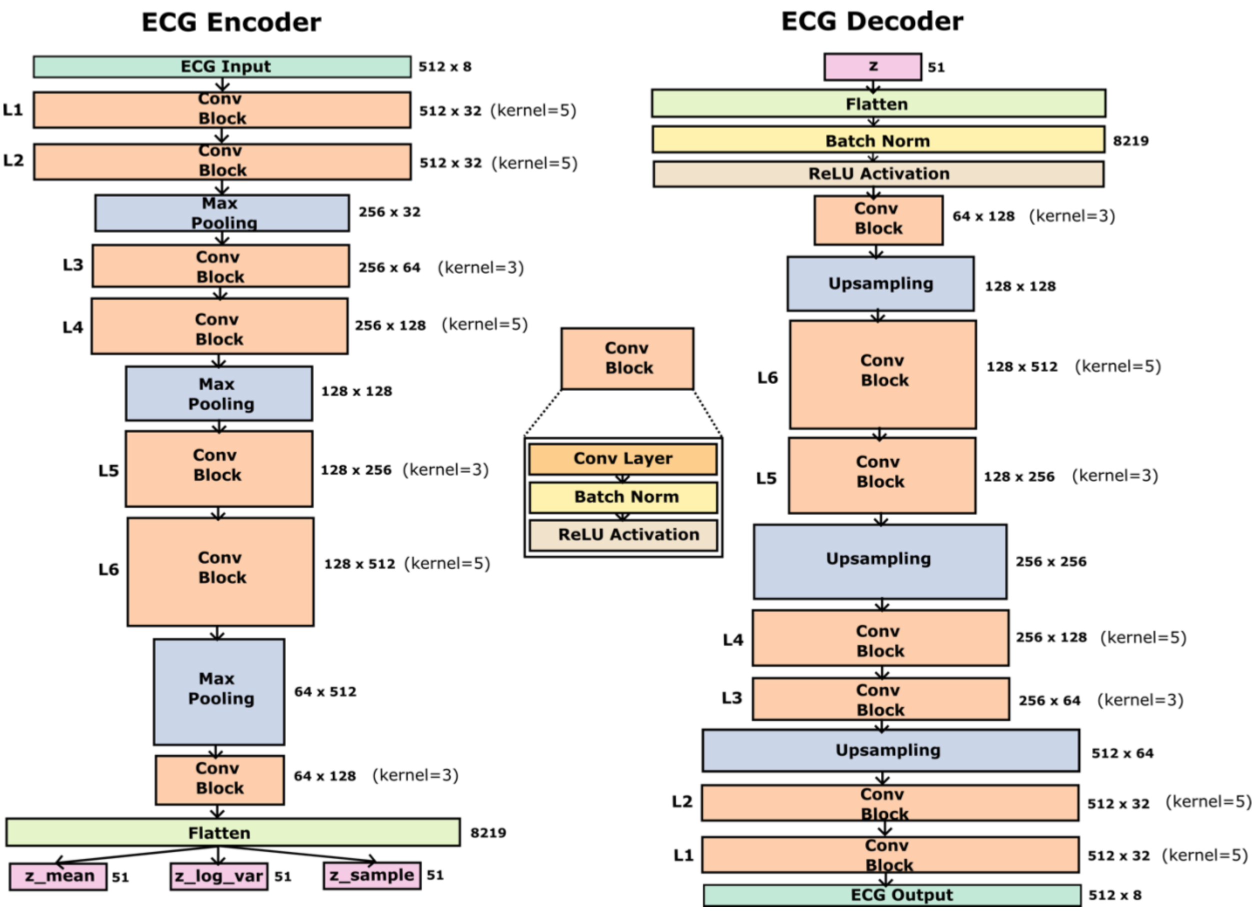

Convolutional encoder and decoder architecture used for the training of the variational autoencoder model.

Supplementary Figure 13

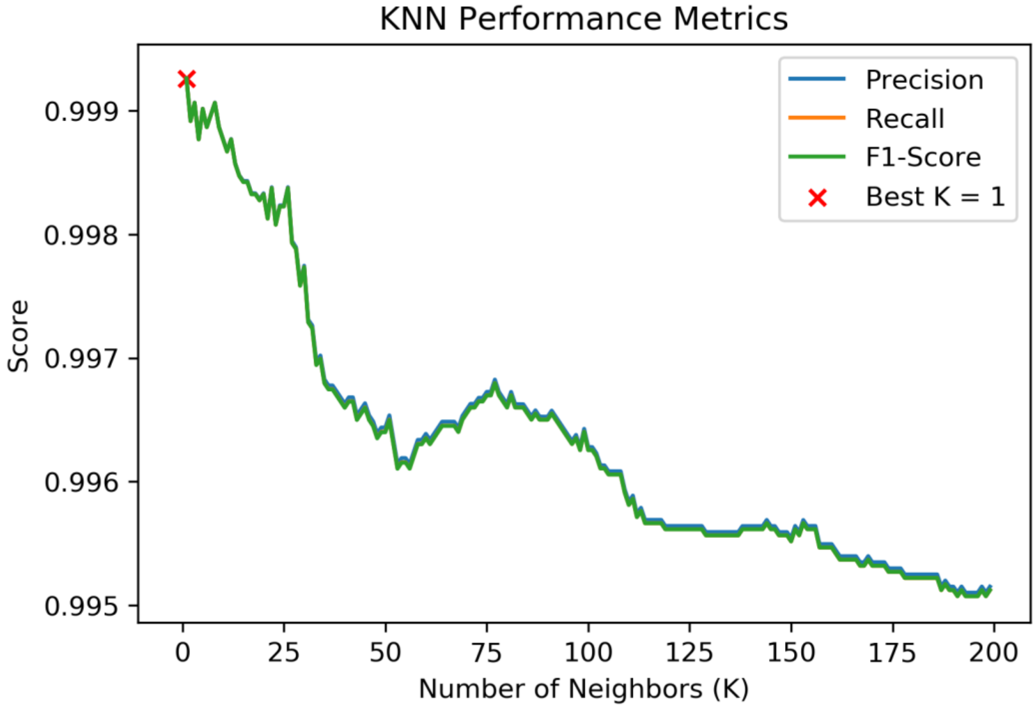

Performance of the K-nearest neighbours (KNN) supervised model in the internal hold-out test set from the BIDMC AF population. Across the different classification metrics (precision, recall, F1-score), the best performance was seen for K of 1. This was used to train a model to predict phenogroup assignments in the UKB AF population.

AF atrial fibrillation; BIDMC Beth Israel Deaconess Medical Centre; UKB UK Biobank.

Supplementary Figure 14

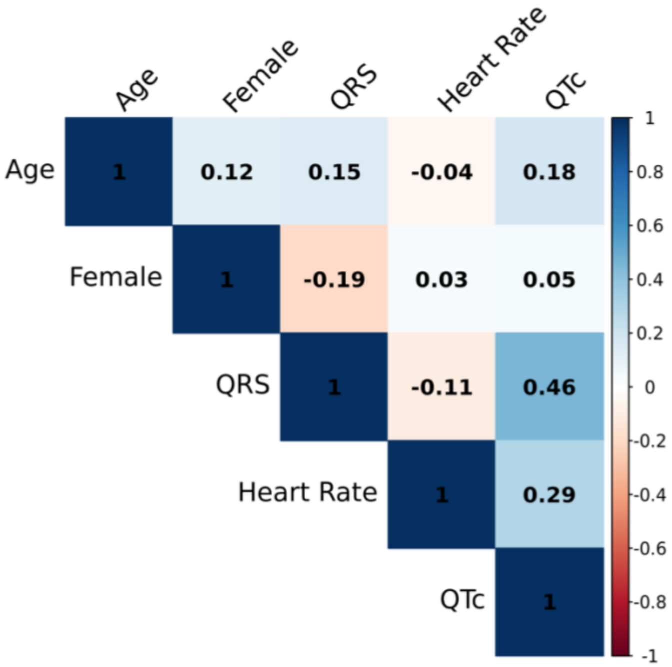

Correlation matrix of covariates included in multivariate logistic models for the BIDMC AF population. *AF atrial fibrillation; BIDMC Beth Israel Deaconess Medical Centre.*
